# Comprehensive safety assessment of Qiwei Tiexie Pill: integrating histopathological, biochemical, and metabolomic analyses in a rat model

**DOI:** 10.3389/fphar.2025.1567316

**Published:** 2025-09-03

**Authors:** Qi Zheng, Wenya Liu, Cong Wang, Zhaoben Kan, Qian Wang, Lu Cui, Zhaoxiang Lin, Feng Zhou, Xin Feng, Junsong Wang

**Affiliations:** ^1^ Center of Molecular Metabolism, Nanjing University of Science and Technology, Nanjing, China; ^2^ Institute for Tibetan Medicine, China Tibetology Research Center, Beijing, China; ^3^ College of Electronics and Information Engineering, South-Central Minzu University, Wuhan, China; ^4^ Technological Innovation Center of Traditional Tibetan Medicine Modernization of XiZang Autonomous Region, Lhasa, China

**Keywords:** Qiwei Tiexie Pill, processed iron powder, metabolomics, hepatotoxicity, nephrotoxicity

## Abstract

**Introduction:**

Qiwei Tiexie Pill (QWTX) is a Tibetan medicine formulation containing processed iron powder that requires systematic safety evaluation. This study aimed to assess the acute toxicological mechanisms of QWTX and its key mineral component, processed iron powder.

**Methods:**

An integrative approach combining histopathological examination, serum biochemistry, and multi-platform metabolomics (1H NMR and LC-MS) was employed to evaluate toxicological responses in Sprague-Dawley rats following 7-day oral administration.

**Results:**

While both treatments preserved hepatic structural integrity without inducing hepatotoxicity, significant renal effects were observed in a dose- and formulation-dependent manner. High-dose processed iron powder caused moderate renal histopathological alterations, primarily vascular changes and hemorrhage, alongside metabolic disruptions in both liver and kidney. QWTX, despite inducing significant metabolic perturbations at high dose, maintained normal renal architecture, indicating a protective effect conferred by its herbal components. Metabolomic and biochemical analyses revealed systemic metabolic reprogramming across four interdependent physiological domains: energy metabolism characterized by TCA cycle impairment and enhanced BCAA catabolism; oxidative stress evidenced by glutathione depletion and lipid peroxidation; nitrogen metabolism showing a “hepato-renal disconnect”; and neuroendocrine regulation with widespread hormone pathway dysregulation.

**Discussion:**

QWTX demonstrated a superior safety profile compared to processed iron powder alone, particularly in preserving renal structure and mitigating iron‐associated nephrotoxicity. However, high‐dose QWTX still triggered significant oxidative and metabolic stress, underscoring the importance of dose optimization in clinical use. These findings provide a systems‐level understanding of the acute toxicological profile of QWTX and processed iron powder, supporting the traditional principles of herbal‐metal synergy in Tibetan medicine while highlighting the need for long-term safety studies to evaluate cumulative mineral exposure and chronic metabolic effects.

## 1 Introduction

Qiwei Tiexie Pill (QWTX), a traditional Tibetan medicine documented in the People’s Republic of China National Pharmacopoeia Commission (2005), includes seven components: four botanical ingredients (*Radix Inulae*, *Costus Root*, *Herba Dracocephali Tangutici*, and *Safflower*), processed iron powder (produced with *Chebulae fructus*), *Gypsum Rubrum* (processed with milk), and *Trogopterus Dung* (Bian et al., 2024). This formulation has been traditionally used to treat hepatic disorders through mechanisms such as promoting qi circulation, enhancing blood flow, regulating liver function, and alleviating pain (Suolang et al., 2019). Clinical evidence supports its efficacy against various liver conditions, including hepatitis B, alcoholic liver disease, fatty liver disease, hepatic fibrosis, and drug-induced liver injury (Suolang et al., 2019; Bian et al., 2024; Wang C. et al., 2024). However, safety concerns remain regarding its mineral components, particularly issues related to hardness and potential heavy metal contamination (Liu et al., 2019).

Minerals have been integral to traditional medicine since ancient times and continue to be utilized today. In Tibetan medical theory, minerals are typically modified during processing through combination with herbs or animal products, differentiating them from their natural state (Feng et al., 2023). This practice allows for enhanced therapeutic effects and reduced toxicity through herb-metal interactions, which facilitate drug delivery to target sites (Liu et al., 2019). The iron-containing ore, processed with *Chebulae fructus* into iron powder, serves as the primary mineral component of QWTX. This processing enhances its therapeutic efficacy: residual organic acids, such as gallic acid, act as antioxidants to prevent iron oxidation and maintain bioactivity (Wang C. et al., 2024), while also promoting the conversion of Fe^3+^ to the more bioavailable Fe^2+^, improving iron absorption and minimizing gastrointestinal irritation.

Despite the therapeutic potential of QWTX, limited research on the safety profile of its processed iron powder has hindered its clinical application. Metabolomics offers a promising approach to address this knowledge gap. As an integral aspect of systems biology, metabolomics enables a comprehensive analysis of small molecule metabolites involved in essential physiological processes (Schmidt et al., 2021; Alarcon-Barrera et al., 2022; Liu et al., 2022). This approach facilitates the detection of biochemical alterations underlying toxicity mechanisms and enhances our understanding of human environmental exposures and their impact on disease pathogenesis (Kim and Kang, 2021). Additionally, it allows for the assessment of metabolic networks by tracking dynamic changes in response to pharmaceutical interventions.

This study presents a systematic safety evaluation of processed iron powder and QWTX using Sprague-Dawley rats over a 7-day treatment period. Our comprehensive assessment includes histological examination through hematoxylin and eosin staining, biochemical parameter analysis, and metabolomics. This multi-faceted approach aims to elucidate the safety profiles of both QWTX and its processed iron powder component, potentially facilitating their broader clinical application.

## 2 Materials and methods

### 2.1 Chemicals and reagents

QWTX was obtained from the China Tibetology Research Center. Biochemical assay kits for superoxide dismutase (SOD), malondialdehyde (MDA), glutathione (GSH), aspartate aminotransferase (AST), alanine aminotransferase (ALT), blood urea nitrogen (BUN) and creatinine (CRE) were purchased from Nanjing Jiancheng Bioengineering Institute. Neutrophil gelatinase-associated lipocalin (NGAL) and cystatin C assay kits were purchased from Wuhan Saipei Biotechnology Co., Ltd. Deuterium oxide (D_2_O, 99.9%), sodium 3-trimethylsilyl-1-(2,2,3,3-^2^H_4_) propionate (TSP), and methanol for NMR analysis were purchased from Sigma Chemical Co. Acetonitrile for NMR was obtained from Guangdong Guanghua Sci-Tech Co. All solvents and reagents were of analytical grade. LC-MS-grade methanol and acetonitrile were acquired from Sigma-Aldrich.

### 2.2 Chemical component analysis of QWTX

1 g of pulverized QWTX was dissolved in 80% methanol and subjected to ultrasonic extraction for 60 min, followed by centrifugation at 13,000 rpm for 15 min 1 mL of the supernatant was collected for LC-MS analysis.

### 2.3 Animal experimental design

Sixty male Sprague-Dawley (SD) rats were maintained under controlled environmental conditions (temperature: 25 °C, relative humidity: 50%, 12/12 h light-dark cycle) with *ad libitum* access to standard food and water. After a 7-day acclimatization period, the rats were randomly assigned to six experimental groups: control group (CK, n = 10) receiving 0.9% saline solution, processed iron powder low-dose group (TL, n = 10, 0.022 g/kg), processed iron powder high-dose group (TH, n = 10, 0.088 g/kg), QWTX low-dose group (FL, n = 10, 1 g/kg), QWTX high-dose group (FH, n = 10, 4 g/kg), and acetaminophen group (APAP, n = 10, 2 g/kg) as positive control. “Processed iron powder” refers to the specially prepared iron material derived from processed iron powder through a traditional processing method involving *Chebulae Fructus*, while “iron powder” is used to denote the unprocessed iron filings.

The dosing rationale was based on the human clinical dose of QWTX (1 g/day, equivalent to 0.0167 g/kg/day for a 60 kg adult), with conversion to rat equivalent doses using body surface area (BSA) normalization. Following ICH S3A and OECD Guideline 407 recommendations for safety evaluation and NOAEL determination, the low and high doses were set at 10- and 40-fold the clinical equivalent dose, respectively. Processed iron powder doses were calculated proportionally based on its content in QWTX.

QWTX and processed iron powder were pulverized and suspended in 0.9% sodium chloride solution containing 0.2% carboxymethylcellulose sodium (CMC-Na). Uniform dispersion was achieved through ultrasonication prior to administration. The control group received an equivalent volume of the vehicle solution. All experimental procedures were conducted in compliance with the guidelines approved by the Chinese Council on Animal Care and were authorized by the Institutional Animal Care and Use Committee at Nanjing University of Science and Technology (Approval ID: ACUC-NUST-20240709).

### 2.4 Sample collection and processing

Following 7 days of treatment and 12 h of fasting, rats were anesthetized for blood collection from the femoral artery, followed by euthanization for liver and kidney harvest. Blood samples were centrifuged (3,000 rpm, 10 min, 4 °C) to obtain serum, which was stored at −80 °C. Harvested organs were divided: one portion was fixed in 4% paraformaldehyde for histopathological examination, while the other was rinsed with cold saline, dried, and stored at −80 °C for ^1^H NMR analysis and LC-MS analysis.

### 2.5 Histopathological analysis and biochemical assessment

Fixed tissue samples were embedded in paraffin, sectioned to 5-μm thickness, and stained with hematoxylin-eosin (H&E) for morphological examination. Semi-quantitative histopathological analysis was performed independently by two experienced pathologists who were blinded to the group allocation. Renal tissue sections were evaluated using the Remuzzi scoring system, while hepatic tissues were assessed according to the Ishak grading system. Serum biochemical parameters were analyzed using commercial assay kits. Oxidative stress markers including superoxide dismutase (SOD), glutathione (GSH), and malondialdehyde (MDA) were measured. Liver function was assessed through alanine aminotransferase (ALT) and aspartate aminotransferase (AST) levels. Kidney function was evaluated by measuring creatinine (CRE), blood urea nitrogen (BUN), neutrophil gelatinase-associated lipocalin (NGAL), and cystatin C levels.

### 2.6 Sample preparation for metabolomics analysis

For NMR analysis, tissue samples (500–600 mg) were homogenized with pre-cooled 50% acetonitrile (1:5 w/v) and centrifuged at 12,000 rpm for 10 min at 4 °C. The supernatant was concentrated under nitrogen stream, stored overnight at −80 °C, and subsequently lyophilized. The dried extracts were reconstituted in 600 μL of D_2_O phosphate buffer (0.2 M, pH 7.0) containing 0.05% TSP as an internal standard.

For LC-MS analysis, tissue samples (60 mg) were homogenized in pre-cooled methanol/water solution (4:1, v/v, 150 μL per 10 mg tissue) using a homogenizer at 70 Hz with three cycles of homogenization-rest (20s–5s) at 4 °C. Proteins were precipitated by incubating the homogenate at −20 °C for 1 h. The mixture was then centrifuged at 16,000 g for 15 min at 4 °C. The supernatant was collected and concentrated under nitrogen stream, followed by overnight storage at −80 °C. The samples were lyophilized using a vacuum freeze-dryer and reconstituted in 150 μL of methanol/water solution (1:1, v/v). After centrifugation at 16,000 g for 15 min at 4 °C to remove debris, the supernatant was transferred to UHPLC vials for LC-MS analysis.

### 2.7 Data acquisition and processing

#### 2.7.1 ^1^H NMR spectroscopy and data analysis

^1^H NMR spectra were acquired on a Bruker AVANCE III 500 MHz spectrometer using CPMG pulse sequence for protein signal suppression. Acquisition parameters included 128 scans, 32,768 data points, 10,000 Hz spectral width, 3.27-s acquisition time, and 3.0-s relaxation delay. Data processing was performed using Bruker TopSpin 3.5 and MestReNova software. The processed spectra were imported into R for multivariate analysis after removing water signals (4.83–5.30 ppm), applying probability quotient normalization and Pareto scaling. Orthogonal signal correction partial least squares discriminant analysis (OSC-PLS-DA) was employed to enhance group discrimination. The statistical significance of the OPLS-DA model’s predictive quality parameters was rigorously assessed using permutation testing (1,000 permutations) and double cross-validation (2CV). Permutation testing evaluates model validity by comparing the predictive ability (Q^2^Y) and goodness-of-fit (R^2^Y) of the model using the true class labels against models generated using randomly permuted class labels. A negative or very low Q^2^Y value indicates a lack of statistically significant group discrimination and potential overfitting. These validation procedures ensure the model’s discriminatory power is robust and not an artifact of overfitting. Variable selection was based on rigorous criteria: metabolites with VIP >1.0 from the OPLS-DA model and p < 0.05 from univariate statistical tests were considered significant. Score plots were generated to visualize group variances, while s-plots and color load diagrams illustrated metabolite contributions to group separation. Metabolite identification was accomplished using HMDB, MMCD databases, STOCSY analysis, and Chenomx NMR Suite v.8.1.

#### 2.7.2 LC-MS analysis and data processing

LC-MS analysis was performed on a hybrid quadrupole time-of-flight mass spectrometer (Triple TOF™ 5600+, AB Sciex) equipped with a Duospray ion source operating in both positive and negative ESI modes. Source parameters were optimized as follows: nitrogen as nebulizer and auxiliary gas, Gas 1 and Gas 2 at 55 psi, curtain gas at 35 psi, ion source temperature at 550 °C, and spray voltage at 5500 V(+)/−4500 V(−). For TOF MS-IDA-MS/MS acquisition, mass ranges were set to 100–1,250 m/z (TOF MS) and 50–1,250 m/z (product ion scan), with accumulation times of 0.10 s and 0.05 s per spectrum, respectively. Information-dependent acquisition was conducted in high-sensitivity mode with optimized parameters including declustering potential at ±80 V, collision energy at 35 ± 15 eV, and dynamic background subtraction activation.

Data were acquired using SCIEX Analyst TF 1.8.1 software. Raw data files (.wiff.scan) were converted to. mzML format using ProteoWizard and processed using XCMS for peak alignment, retention time correction, and peak area extraction. Metabolite identification was achieved through accurate mass matching (<25 ppm) and MS/MS spectral matching against databases. Data normalization was performed using Pareto-scaling, followed by multivariate statistical analysis using R software, including partial least squares discriminant analysis (PLS-DA). Univariate statistical analyses comprised t-tests and fold change analysis, with results visualized through heatmaps generated in R.

### 2.8 KEGG pathway analysis and enrichment ananlysis

We performed KEGG pathway analysis and enrichment analysis on the identified differential metabolites between the treatment and control groups using MetaboAnalyst 6.0 (https://www.metaboanalyst.ca/).

### 2.9 Quantitative real-time PCR

Total RNA was extracted from samples using TRIzol™ Reagent (Gibco, Grand Island, NY, USA) following the manufacturer’s protocol. Complementary DNA (cDNA) was synthesized from the extracted RNA via reverse transcription using a commercial reverse transcriptase kit. Quantitative real-time PCR (qRT-PCR) was subsequently performed using the KAPA SYBR FAST qPCR Kit (KAPA Biosystems, Beijing, China) on a 7,300 Real-Time PCR System (Applied Biosystems). β-actin served as the internal control and reference gene for normalization of target gene expression. All gene-specific primers used in this study were synthesized by Tsingke Biotechnology Co., Ltd. (Nanjing, China), and their sequences are provided in Supplementary Table S1.

### 2.10 Statistical analysis

Statistical analysis was performed using GraphPad Prism 9.0 (GraphPad Software, United States) and R software. Statistical significance was assessed using Student’s t-tests or Mann-Whitney tests with Benjamin-Hochberg correction for multiple comparisons. Significance was set at p < 0.05, and results were expressed as fold-change (FC) values relative to controls.

## 3 Result

### 3.1 Chemical composition analysis of QWTX

LC-MS analysis identified 36 compounds in QWTX. Notable components included organic acids such as ellagic acid, gallic acid, gallic acid-3-o-(6′-o-galloyl) glucoside, and gallotannin, which may contribute to the antioxidant effects of processed iron powder. Additionally, anti-inflammatory compounds including ellagic acid, gallic acid, gentiopicrin, gallotannin, and apocynin were identified, suggesting potential mechanisms for the formula’s therapeutic effects in liver diseases (Supplementary Table S2).

### 3.2 Evaluation of histopathological effects of QWTX and processed processed iron powder in rat tissues

**FIGURE 1:**
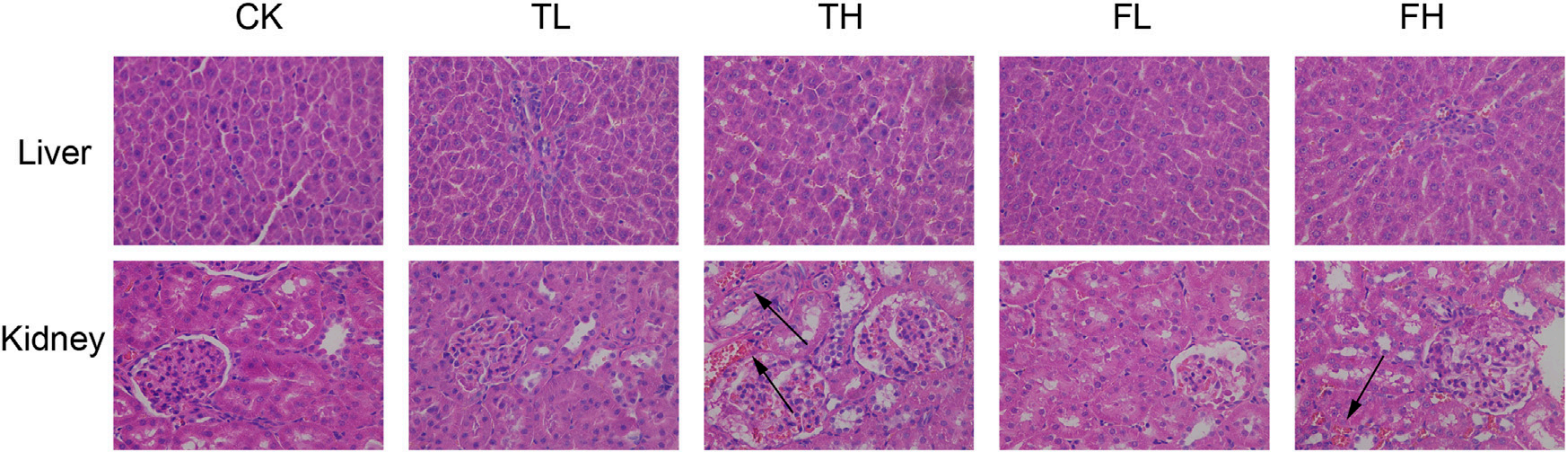
Representative histopathological images of rat liver and kidney tissues stained with hematoxylin and eosin (H&E, ×400 magnification). Groups: control (CK), low-dose processed iron powder (TL), high-dose processed iron powder (TH), low-dose QWTX (FL), and high-dose QWTX (FH). black arrows indicate tissue hemorrhage.

Histopathological examination revealed tissue-specific responses to the treatments (Figure 1). Semi-quantitative analysis of liver tissues using the Ishak scoring system showed that all treatment groups (TL, TH, FL, and FH) maintained normal hepatic architecture similar to the control group (CK), with scores of 0 for fibrosis, inflammatory cell infiltration, necrosis, and structural changes.

In kidney tissues, Remuzzi scoring revealed significant differences among treatment groups (Figure 1). The control group (CK) and low-dose groups (TL and FL) showed minimal pathological changes (scores <1). However, the high-dose processed iron powder group (TH) showed significantly elevated scores (2.3 ± 0.4, p < 0.01 vs. CK), primarily due to vascular changes and hemorrhage. The high-dose QWTX group (FH) maintained relatively normal renal structure with scores comparable to the control group. These findings indicate that while low doses maintain tissue integrity, high-dose processed iron powder may induce moderate renal pathological changes.

### 3.3 Biochemical assessment of oxidative stress and organ function following QWTX and processed iron powder treatment

**FIGURE 2:**
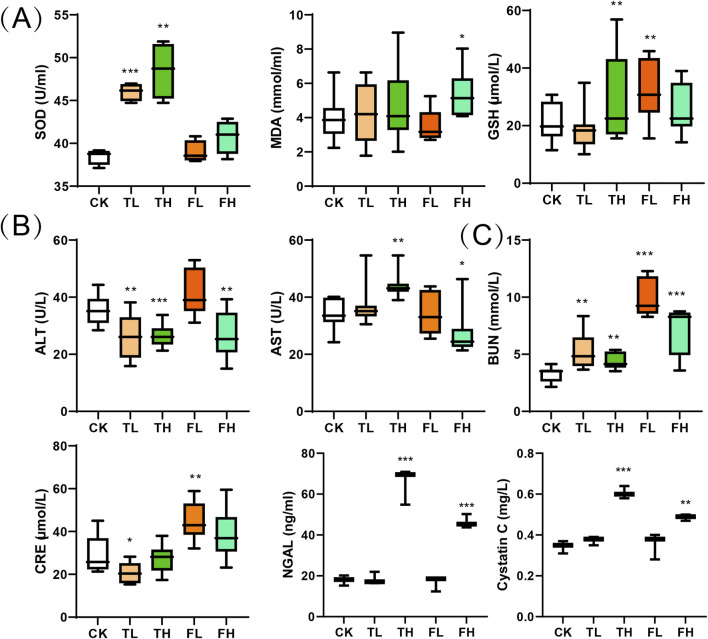
Biochemical parameters across treatment groups presented as box plots. **(A)** Oxidative stress markers: SOD, MDA, and GSH. **(B)** Liver function indicators: ALT and AST. **(C)** Kidney function markers: BUN, CRE, NGAL and cystatin C. Boxes show interquartile ranges (25th-75th percentiles) with median lines; whiskers extend to minimum and maximum values within 1.5 times the interquartile range; outliers are shown as individual points. Data represent mean ± SD (n = 3). Statistical significance versus control group: *p < 0.05, **p < 0.01, ***p < 0.001.

Analysis of oxidative stress markers revealed distinct treatment-dependent responses (Figure 2A). Superoxide Dismutase (SOD) activity significantly increased in both the low-dose (TL) and high-dose (TH) groups compared to the control group (CK). Glutathione (GSH) levels were significantly elevated in the TH and low-dose QWTX (FL) groups, indicating enhanced antioxidant capacity. In contrast, Malondialdehyde (MDA) levels significantly increased in the high-dose QWTX group (FH), suggesting heightened lipid peroxidation and oxidative stress.

Liver function parameters showed variable responses across treatment groups (Figure 2B). ALT levels significantly decreased in the TL, TH, and FH groups compared to CK, indicating improved liver function. Conversely, AST levels were significantly elevated in the TH group but decreased in the FH group, demonstrating differential effects on hepatic function.

Renal function markers demonstrated dose-dependent alterations across treatment groups (Figure 2C). Blood Urea Nitrogen (BUN) levels increased significantly in all treated cohorts, with modest elevation in TL and TH groups versus substantial increases in FL and FH. Creatinine (CRE) exhibited divergent responses: decreased in TL (p < 0.05) but significantly increased in FL. Furthermore, both NGAL and Cystatin C showed marked elevation in high-dose groups (FH, TH) compared to controls (CK), indicating potential nephrotoxicity.

### 3.4 Metabolomic profiling reveals distinct effects of processed iron powder and QWTX on rat liver and kidney metabolism

**FIGURE 3:**
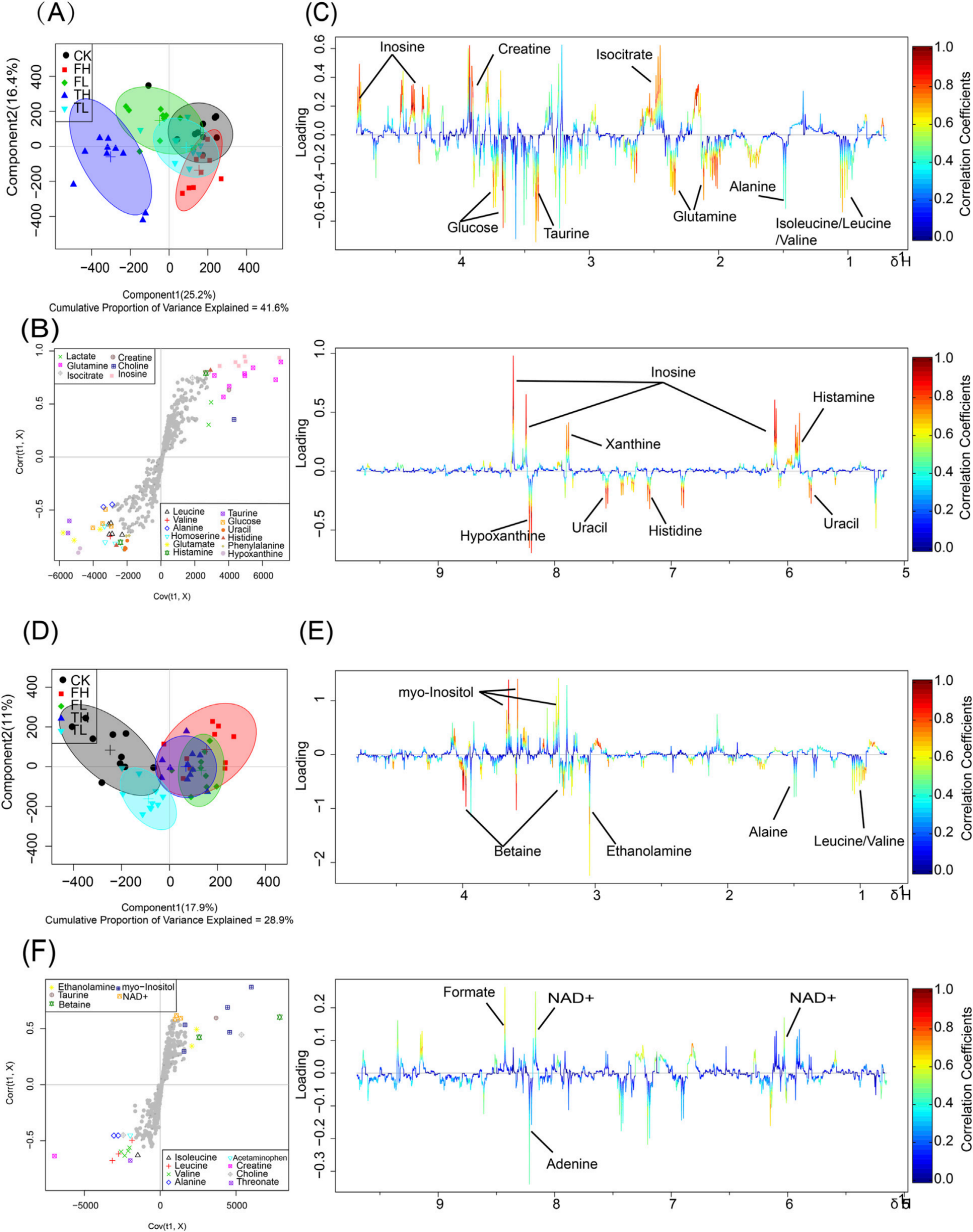
OPLS-DA analysis and metabolic pathway enrichment based on NMR data from liver and kidney tissues following administration of processed iron powder and QWTX. Score plots, S-plots, and color-coded loading plots for liver **(A–C)** and kidney **(D–F)** tissues. The score plots **(A,D)** show group separation patterns, while S-plots **(B,E)** and color-coded loading plots **(C,F)** identify metabolites contributing to group discrimination.

Metabolomic profiling using NMR revealed significant metabolic differences among experimental groups. To enhance the differentiation between groups, OPLS-DA was utilized to filter out irrelevant variables and elucidate overall metabolic patterns. In the liver score plot (Figure 3A), the TH group showed complete separation from the CK group, indicating that high-dose processed iron powder induced metabolic disorders in rat liver (R^2^Y = 0.87, Q^2^ = 0.75, *p* = 0.026). The QWTX groups (both high and low doses) largely overlapped with the CK group, suggesting their overall metabolic profiles were similar to the control. In kidney tissue (Figure 3D), the FL group demonstrated complete separation from the CK group, indicating metabolic perturbation in kidney tissue (R^2^Y = 0.81, Q^2^ = 0.7, *p* = 0.03). Additionally, both FH and TH groups showed slight separation from the CK group, suggesting potential metabolic toxicity.

The S-plots and color-coded loading plots revealed the metabolites that contributed significantly to group separation. In liver tissue, significant contributors included isoleucine, leucine, valine, alanine, glutamine, isocitrate, taurine, glucose, creatine, inosine, uracil, histidine, xanthine, and hypoxanthine (Figures 3B,C). In kidney tissue, the main contributing metabolites were leucine, valine, alanine, ethanolamine, betaine, myo-inositol, adenine, and formate (Figures 3E,F).

**FIGURE 4:**
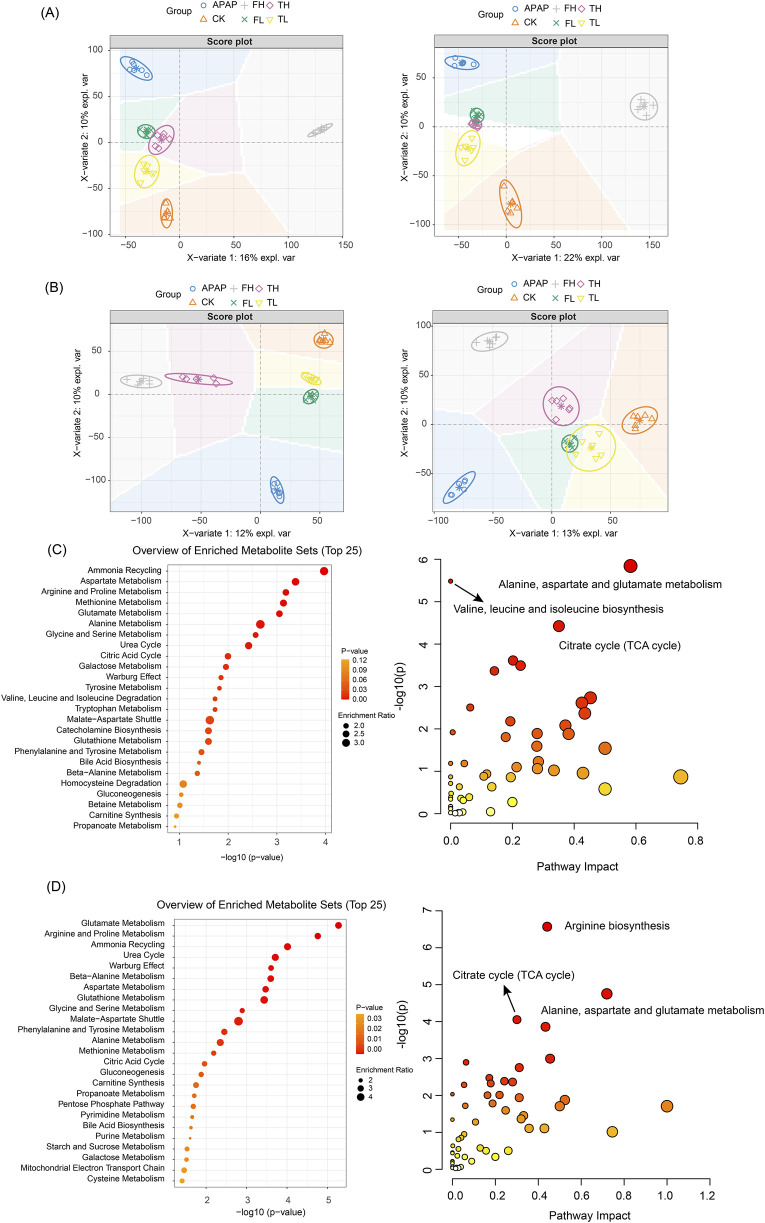
OPLS-DA analysis and metabolic pathway enrichment based on LC-MS data from liver and kidney tissues following administration of processed iron powder and QWTX. **(A)** Liver tissue OPLS-DA score plot (R^2^Y = 0.95, Q^2^ = 0.85); **(B)** The left panel shows the anion mode, and the right panel shows the cation mode; **(C)** Liver metabolic pathway enrichment (Top 25); **(D)** Kidney metabolic pathway enrichment (Top 25). Left panels: Pathways ranked by -log_10_(p-value). Right panels: Pathway impact versus enrichment ratio plot.

In liver tissues (Figure 4A), the OPLS-DA model demonstrated robust statistical reliability (R^2^Y = 0.95, Q^2^ = 0.85, *p* = 0.02) in both negative and positive ion modes. Along the first principal component (PC1), which explained 16%–22% of the total variance, the high-dose QWTX group (FH) showed the greatest separation from the control group (CK), suggesting that QWTX treatment induced the most substantial alterations in hepatic metabolism. Along PC2 (10% explained variance), the APAP group exhibited maximal deviation from other groups, particularly in the negative ion mode, indicating APAP-induced metabolic perturbations distinct from those caused by either processed iron powder or QWTX treatments.

In kidney tissues (Figure 4B), the OPLS-DA model (R^2^Y = 0.96, Q^2^ = 0.73, *p* = 0.016) revealed treatment-specific clustering patterns. In the negative ion mode (left panel, PC1: 12% explained variance), the high-dose groups (TH and FH) showed clear separation from the control group, with the APAP group displaying the most pronounced deviation along PC2 (10% explained variance). The positive ion mode analysis (right panel, PC1: 13% explained variance) revealed an interesting pattern where the FH and APAP groups deviated in opposite directions along PC2, suggesting fundamentally different mechanisms of kidney metabolism modulation between QWTX and APAP treatments.

Notably, the low-dose groups (TL and FL) consistently clustered closer to the control group in both tissue types and ionization modes, indicating dose-dependent metabolic effects. These comprehensive metabolomic findings highlight the distinct biological impacts of processed iron powder and QWTX on hepatic and renal metabolism, providing valuable insights into their therapeutic mechanisms and potential toxicological profiles.

### 3.5 Metabolic pathway analysis reveals distinct effects of processed iron powder and QWTX on rat liver and kidney metabolism

**FIGURE 5:**
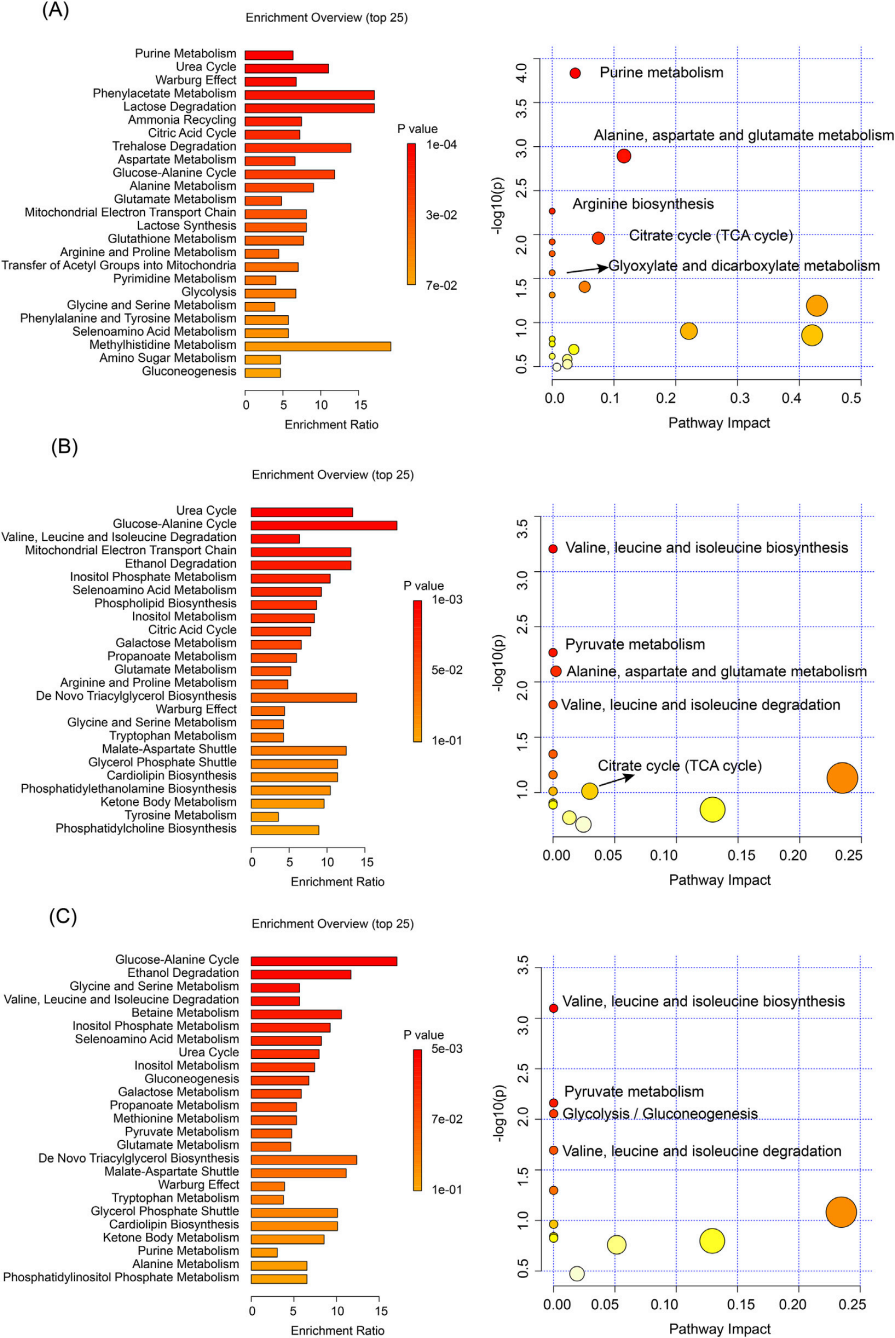
Pathway enrichment and KEGG analyses based on NMR data of differential metabolites. **(A)** Liver tissue analysis comparing TH and CK groups. **(B)** Kidney tissue analysis comparing TH and CK groups. **(C)** Kidney tissue analysis comparing QWTX treatment groups (FL and FH) with CK group. Left panels show metabolic pathway enrichment analysis based on the SMPDB database (bar charts, displaying enrichment ratio and p-values). Right panels display KEGG pathway analysis (bubble plots), where bubble size represents pathway impact and color intensity indicates statistical significance.

The metabolomic analyses using both NMR (Figure 5) and LC-MS technologies (Figures 4C,D) revealed significant metabolic alterations in liver and kidney tissues following processed iron powder and QWTX treatments. The results from both analytical platforms showed remarkable consistency in identifying key affected metabolic pathways, with the integration of pathway impact values and p-values enabling prioritization of biologically relevant disruptions.

In the liver, both techniques demonstrated significant enrichment of amino acid metabolism pathways, particularly involving branched-chain amino acids (BCAAs: valine, leucine, isoleucine), glutamate, and aspartate metabolism. These findings suggest impaired nitrogen shuttling and energy metabolism, potentially linked to mitochondrial dysfunction and oxidative stress. The TCA cycle and energy metabolism were profoundly impacted, with LC-MS additionally identifying Warburg effect-related changes, indicating a shift toward anaerobic glycolysis despite mitochondrial impairment. Notably, both platforms highlighted the enrichment of the urea cycle and nitrogen metabolism pathways, suggesting altered protein metabolism and ammonia detoxification, which may contribute to systemic hyperammonemia or hepatic encephalopathy under chronic exposure.

In kidney tissues, both NMR and LC-MS analyses identified significant changes in amino acid metabolism, particularly in the degradation of BCAAs, reflecting potential disruptions in inter-organ nitrogen balance and energy homeostasis. Energy metabolism pathways were significantly affected, with NMR showing alterations in the glucose-alanine cycle and LC-MS revealing changes in the mitochondrial electron transport chain (ETC), particularly at Complex II. These findings point to renal-specific metabolic reprogramming driven by mitochondrial dysfunction and compensatory gluconeogenesis. The glutathione metabolism pathway was significantly enriched in both tissues, indicating a systemic oxidative stress response, as evidenced by elevated GSSG and cysteine levels.

Importantly, both analytical approaches revealed tissue-specific metabolic responses to processed iron powder and QWTX treatments. In the liver, processed iron powder predominantly affected purine metabolism and ammonia recycling, while QWTX mainly influenced the glucose-alanine cycle and ethanol degradation. In kidney tissues, processed iron powder primarily impacted the urea cycle and mitochondrial ETC, whereas QWTX significantly affected BCAA metabolism and glycine-serine metabolism, indicating differential effects on renal nitrogen handling and one-carbon metabolism.

These findings collectively demonstrate that processed iron powder and QWTX treatments induce comprehensive metabolic reprogramming in both liver and kidney tissues, particularly affecting amino acid metabolism, energy metabolism, and nitrogen metabolism pathways.

### 3.6 Metabolomic profiling reveals tissue-specific alterations across core metabolic domains

**FIGURE 6:**
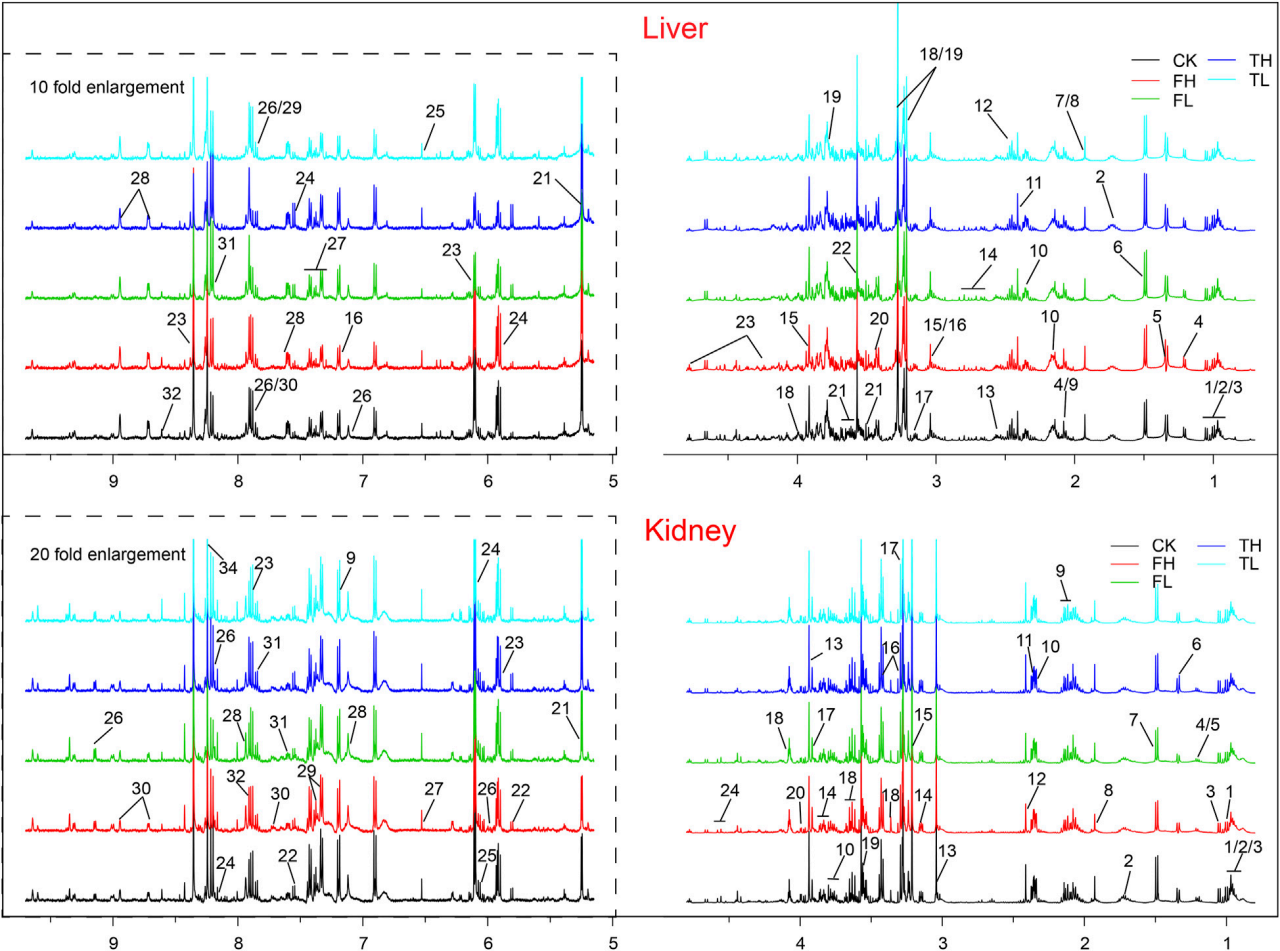
Representative 500 MHz ^1^H NMR spectra of rat liver and kidney extracts showing identified metabolites. Metabolites are numbered and labeled in both liver (top panel) and kidney (bottom panel) spectra. Liver metabolites: 1, Isoleucine; 2, Leucine; 3, Valine; 4, 3-Hydroxybutyrate; 5, Lactate; 6, Alanine; 7, Arginine; 8, Acetate; 9, Homoserine; 10, Glutamine; 11, Glutamate; 12, Succinate; 13, Isocitrate; 14, Glutathione; 15, Creatine; 16, Histamine; 17, Ethanolamine; 18, Choline; 19, Betaine; 20, Taurine; 21, Glucose; 22, Glycine; 23, Inosine; 24, Uracil; 25, Fumarate; 26, Histidine; 27, Phenylalanine; 28, Nicotinurate; 29, Uridine; 30, Xanthine; 31, Hypoxanthine; 32, ATP. Kindney metabolites: 1, Isoleucine; 2, Leucine; 3, Valine; 4, Ethanol; 5, 3-Hydroxybutyrate; 6, Lactate; 7, Alanine; 8, Acetate; 9, Acetaminophen; 10, Glutamate; 11, Succinate; 12, Glutamine; 13, Creatine; 14, Ethanolamine; 15, Choline; 16, Taurine; 17, Betaine; 18, myo-Inositol; 19, Glycine; 20, Threonate; 21, Glucose; 22, Uracil; 23, Uridine; 24, Inosine; 25, Adenosine; 26, NAD+; 27, Fumarate; 28, Histamine; 29, Phenylalanine; 30, Niacinamide; 31, Tryptophan; 32, Xanthine; 33, Adenine; 34, Formate. CK: control group; TL: low-dose processed iron powder group; TH: high-dose processed iron powder group; FL: low-dose QWTX group; FH: high-dose QWTX group.

**FIGURE 7:**
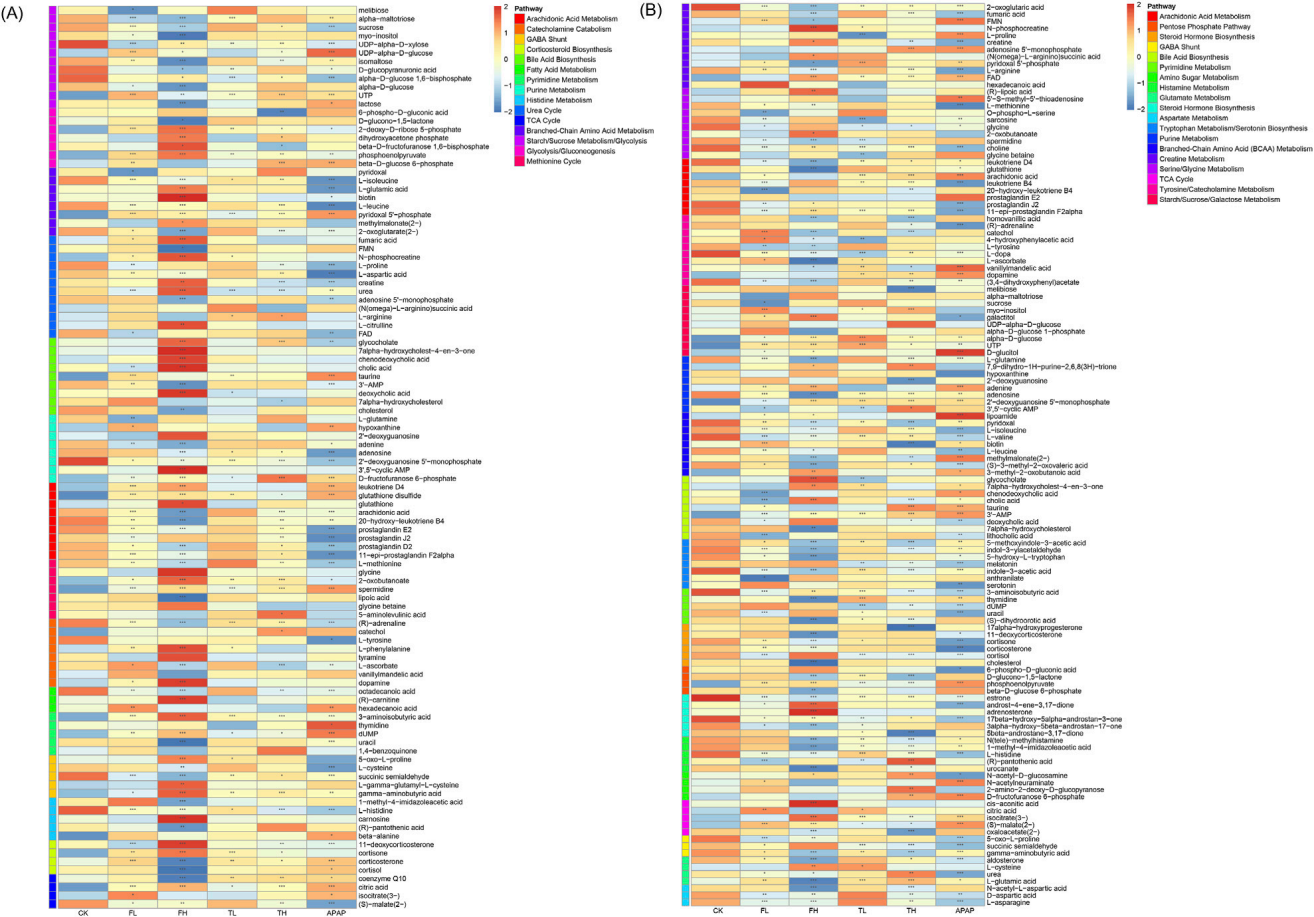
Heatmap showing differentially expressed metabolites in rat liver **(A)** and kidney **(B)** tissues as determined by LC-MS analysis. Metabolites were classified according to metabolic pathways annotated in the SMPDB database. Data are presented as mean ± SD (n = 6). Statistical significance compared to the control group: *p < 0.05, **p < 0.01, ***p < 0.001.

Comprehensive metabolomic profiling, integrating ^1^H NMR spectroscopy and LC-MS platforms, identified significant metabolic reprogramming in liver and kidney tissues. NMR quantified 32 and 30 distinct metabolites in liver and kidney, respectively (Tables 1, 2), while LC-MS detected 107 and 130 differentially expressed metabolites in these tissues, ensuring broad coverage and analytical robustness (Figures 6, 7). In addition, the significantly altered metabolites from LC-MS were classified into different metabolic pathways according to the SMPDB database.

**TABLE 1:** Identified metabolites in the liver from different groups with log_2_(FC) and p-values.

Metabolites	TL vs. CK		TH vs. CK		FL vs. CK		FH vs. CK	
	Log_2_(FC)	*P*	Log_2_(FC)	*P*	Log_2_(FC)	*P*	Log_2_(FC)	*P*
Isoleucine	0.13		0.15		0.09		−0.15	
Leucine	0.01		0.21		0.16		−0.15	
Valine	−0.01		0.19		0.19		−0.14	
3-Hydroxybutyrate	−0.06		−0.03		−0.08		0.03	
Lactate	0.08		−0.13		0.02		0.27	
Alanine	0.09		0.2	*	0.08		−0.02	
Arginine	−0.08		0.1		−0.12		−0.25	
Acetate	0.15		−0.01		−0.03		−0.08	
Homoserine	0.07		0.27		0.15		−0.12	
Glutamine	−0.05		−0.22	***	−0.08		0.12	
Glutamate	−0.02		0.33		0.16		−0.09	
Succinate	−0.08		0.21		−0.06		−0.04	
Isocitrate	−0.08		−0.22	*	−0.13		0.13	
Glutathione	−0.04		−0.27		−0.18		0.04	
Creatine	0.06		−0.41	*	0.07		−0.19	
Histamine	−0.06		0.11		0.2	*	−0.14	
Ethanolamine	−0.14		0.1		−0.02		−0.09	
Choline	0		−0.16		−0.07		−0.03	
Betaine	0.03		−0.05		0.24		−0.16	
Taurine	0.26		0.55	*	0.04		0.1	
Glucose	0.28		0.27	*	0.06		0.2	
Glycine	0.06		0.19		0.35		0.11	
Inosine	−0.07		−1.31	***	−0.92	**	0.03	
Uracil	0.39		1.44	***	0.86	*	0.2	
Fumarate	0.16		0.38	*	−0.01		0.28	
Histidine	0.13		−0.22	*	−0.02		−0.04	
Phenylalanine	−0.03		0.27		−0.09		−0.17	
Nicotinurate	−0.04		−0.21		−0.13		0.01	
Uridine	0.06		0.06		−0.25		−0.09	
Xanthine	0.41		0.74	*	0.68		0.18	
Hypoxanthine	0.08		1.02	***	0.85	**	0.11	
ATP	−0.2		−0.24	*	−0.18		0.34	

Log_2_(FC) values are represented by color intensity: red indicates increased levels and blue indicates decreased levels, with deeper colors representing greater magnitude of change. Statistical significance: *p < 0.05, **p < 0.01, and ***p < 0.001.

**TABLE 2:** Identified metabolites in the kidney from different groups with log_2_(FC) and p-values.

Metabolites	TL vs. CK		TH vs. CK		FL vs. CK		FH vs. CK	
	Log_2_(FC)	*p*	Log_2_(FC)	*p*	Log_2_(FC)	*p*	Log_2_(FC)	*p*
Isoleucine	−0.01		−0.01		0.02		−0.03	
Leucine	−0.34		−0.39	*	−0.33	*	−0.34	*
Valine	−0.24		−0.26	*	−0.26	*	−0.27	*
Ethanol	−0.5		−0.44	*	−0.4		−0.53	**
3-Hydroxybutyrate	−0.1		−0.09		−0.16		−0.06	
Lactate	−0.46		0.02		−0.79	*	−0.39	
Alanine	−0.33		−0.29	*	−0.28	*	−0.27	*
Acetate	−0.12		0.04		−0.14		−0.01	
Acetaminophen	−0.12		−0.16	*	−0.13		−0.1	
Glutamate	0		−0.02		−0.01		−0.05	
Succinate	0.08		0.04		−0.06		−0.03	
Glutamine	−0.1		−0.01		−0.07		−0.13	
Creatine	−0.05		−0.14		−0.22		−0.14	
Ethanolamine	0.01		0.15	*	0.1		0.09	
Choline	0		0.06		0.01		0.1	
Taurine	0.07		0.08		0.02		0.02	
Betaine	0.13		0.1		0.3	**	0.32	**
Myo-Inositol	0.25		0.25	*	0.31	**	0.3	**
Glycine	−0.08		−0.08		−0.09		0.07	
Threonate	−0.05		−0.29		−0.29	*	−0.44	**
Glucose	0.42		0.29		−0.02		0.04	
Uracil	−0.08		0.18		0.02		−0.08	
Uridine	0.11		−0.01		0.17		0.01	
Inosine	0.19		0.07		0.09		−0.05	
Adenosine	0.12		0.13		0.11		0.08	
NAD+	0.36		0.35	*	0.78	***	0.39	*
Fumarate	0.34		0.25	*	0.19		0.18	
Histamine	0.06		−0.42		0.06		−0.07	
Phenylalanine	−0.06		−0.29		−0.22		−0.17	
Niacinamide	−0.17		−0.02		−0.06		0.01	
Tryptophan	−0.02		0.01		0.03		0	
Xanthine	−0.2		0.15		−0.26		0.02	
Adenine	−0.27		−0.26		−0.5	*	−0.25	
Formate	−0.13		0.27		−0.08		0.14	

Log_2_(FC) values are represented by color intensity: red indicates increased levels and blue indicates decreased levels, with deeper colors representing greater magnitude of change. Statistical significance: *p < 0.05, **p < 0.01, and ***p < 0.001.

In liver tissue, perturbed pathways were stratified as follows: Energy metabolism pathways exhibited pronounced alterations, including core bioenergetic hubs (TCA Cycle, Glycolysis/Gluconeogenesis, Pentose Phosphate Pathway, Fatty Acid Oxidation) and substrate-processing routes (Glycogen Metabolism, Starch/Sucrose Metabolism, Galactose Metabolism, Branched-Chain Amino Acid Metabolism). Oxidative stress responses were dominated by redox defense systems (Arachidonic Acid Metabolism, Glutathione Metabolism, Lipoic Acid Metabolism, Pentose Phosphate Pathway—via NADPH supply for glutathione reduction), alongside cofactor-dependent mechanisms (Riboflavin Metabolism, Vitamin B6 Metabolism) (Moini et al., 2002). Nitrogen cycle metabolism encompassed nitrogen handling (Urea Cycle), amino acid/nucleotide catabolism (Purine/Pyrimidine Metabolism, Histidine/Tyrosine Metabolism, Polyamine Metabolism), and methyl-group transfer (Methionine Cycle). Critically, neuro-endocrine pathways showed marked dysregulation, particularly in lipid-derived signaling (Arachidonic Acid Metabolism—prostanoid synthesis), neurotransmitter systems (GABA Shunt, Catecholamine Biosynthesis, Serotonin Biosynthesis), and steroid hormone networks (Corticosteroid Biosynthesis, Bile Acid Biosynthesis).

In kidney tissue, distinct patterns emerged: Energy metabolism alterations centered on carbohydrate flux (TCA Cycle, Glycolysis/Gluconeogenesis, Pentose Phosphate Pathway, Polyol Pathway, Amino Sugar Metabolism) and osmolyte-linked pathways (Inositol Metabolism, Nucleotide Sugar Metabolism). Oxidative stress defenses prominently featured glutathione-centric networks (Glutathione Metabolism, Cysteine and Methionine Metabolism) and precursor pathways (Serine/Glycine Metabolism, Glycine Metabolism), augmented by osmoprotectant systems (Osmolyte Accumulation) and B-vitamin cofactors (Riboflavin/Vitamin B6 Metabolism). Nitrogen metabolism was characterized by extensive amino acid processing (Branched-Chain Amino Acid Metabolism, Arginine and Proline Metabolism, Glutamate/Aspartate Metabolism) and nucleotide-related pathways (Purine/Pyrimidine Metabolism, Creatine Metabolism), with Phosphatidylcholine Metabolism linking methyl metabolism to nitrogen handling. Neuro-endocrine disruptions involved neurotransmitter synthesis (Tyrosine/Tryptophan Metabolism for catecholamines/serotonin, Histamine Metabolism) and steroid-derived signaling (Steroid/Corticosteroid Biosynthesis, Bile Acid Biosynthesis with Taurine Metabolism).

This domain-specific stratification demonstrates that metabolic reprogramming extends beyond isolated pathways to coordinated dysregulation across four fundamental physiological axes: bioenergetic homeostasis (energy metabolism), redox balance (oxidative stress), nitrogen handling (nitrogen cycle metabolism), and intercellular communication (neuro-endocrine system), with liver emphasizing steroid/neurotransmitter signaling and kidney highlighting osmolyte-linked redox and nitrogen processing.

### 3.7 QPCR validation reveals gene expression changes in liver and kidney tissues

**FIGURE 8:**
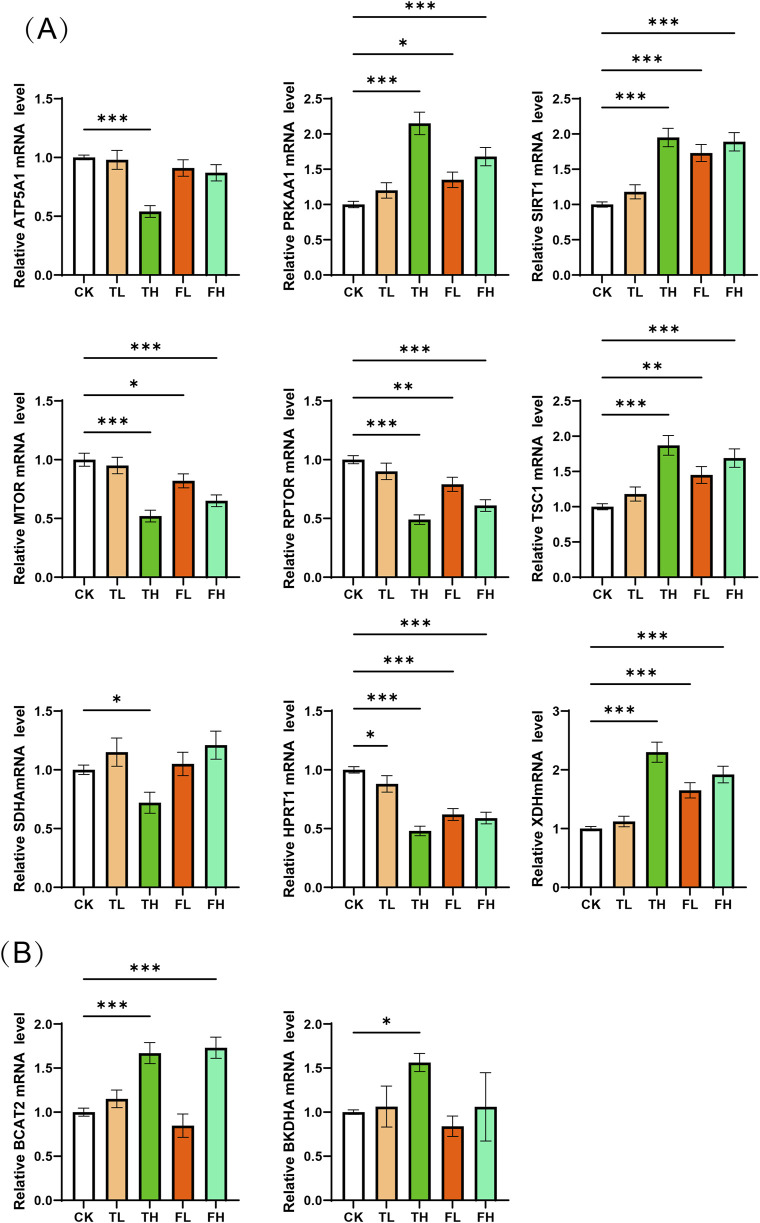
qPCR validation of mRNA expression levels for key enzymes. **(A)** Relative mRNA expression of key enzyme genes in liver tissue across experimental conditions. Genes analyzed: ATP5A1, PRKAA1, SIRT1, MTOR, RPTOR, TSC1, SDHA, HPRT1, XDH. **(B)** Relative mRNA expression of key enzyme genes in kidney tissue across experimental conditions. Genes analyzed: BCAT2, BCKDHA. Data represent mean ± SD (n = 3). Statistical significance versus control group: *p < 0.05, **p < 0.01, ***p < 0.001.

qPCR validation revealed significant gene expression alterations in liver and kidney tissues (Figure 8). In the liver, ATP5A1, MTOR, RPTOR, SDHA, and HPRT1 were markedly downregulated in TH group compared to the CK group, while PRKAA1, SIRT1, and XDH were significantly upregulated. In kidney tissue, BCAT2 and BCKDHA exhibited significant upregulation in TH relative to CK. These transcriptional changes are consistent with and support the metabolomic findings, suggesting coordinated molecular responses underlying the observed metabolic shifts.

## 4 Discussion

Although QWTX has demonstrated therapeutic efficacy in the treatment of liver diseases, its safety profile remains poorly characterized. Of particular concern is the presence of processed iron powder, a metallic component that raises potential safety issues and may limit its clinical application. To address this gap, the present study employed a comprehensive approach integrating histopathological examination, serum biochemistry, and metabolomic profiling to assess the potential toxicity of both processed iron powder and QWTX in Sprague-Dawley (SD) rats following 7 days of oral administration.

### 4.1 Integrated toxicological assessment of processed iron powder and QWTX

Histopathological and biochemical analyses revealed distinct, tissue-specific responses to both processed iron powder and QWTX, highlighting complex patterns of organ response and differential toxicity potential (Figure 1). In the liver, the preservation of normal histological architecture across all treatment groups, coupled with significant reductions in serum alanine aminotransferase (ALT) levels in the TL, TH, and FH groups, strongly indicates maintained hepatic integrity (Figure 2). ALT is predominantly localized in hepatocyte cytoplasm, where its concentration is approximately 3,000-fold higher than in serum, making it a highly sensitive and specific biomarker of hepatocellular injury (Thakur et al., 2024). The observed decrease in ALT levels thus provides compelling evidence that both processed iron powder and QWTX do not induce hepatotoxicity and may even support liver health under the tested conditions (Li et al., 2024).

Although aspartate aminotransferase (AST) levels were elevated in the TH group, this finding must be interpreted with caution. AST is not liver-specific and is abundantly expressed in the heart, skeletal muscle, kidneys, and other tissues (Ndrepepa and Medicine, 2020). Therefore, increased AST may reflect systemic metabolic or muscular effects rather than direct liver damage. This interpretation is strongly supported by the absence of histopathological abnormalities in the liver and the lack of concomitant elevation in other liver-specific markers, such as ALT or bilirubin, in the TH group.

In contrast, renal tissue exhibited more nuanced and dose-dependent responses. Low-dose treatments (TL and FL) preserved normal renal architecture, with Remuzzi scores below 1, indicating minimal pathology. However, the high-dose processed iron powder group (TH) displayed significant renal damage (Remuzzi score: 2.3 ± 0.4), characterized primarily by vascular alterations and focal hemorrhage—findings consistent with iron-induced oxidative stress (Chen et al., 2021). Notably, high-dose QWTX (FH group) conferred renal protection, with histological structure comparable to controls despite elevated levels of oxidative stress markers such as malondialdehyde (MDA). This suggests that components of QWTX may mitigate iron-associated nephrotoxicity, potentially through antioxidant or cytoprotective mechanisms.

### 4.2 Metabolomic profiling of QWTX and processed iron powder reveals tissue-specific metabolic perturbations and distinct mechanisms of action

This study employed an integrated metabolomics approach combining ^1^H NMR and LC-MS to comprehensively characterize treatment-induced metabolic alterations in hepatic and renal tissues of rats. Orthogonal partial least squares-discriminant analysis (OPLS-DA) revealed distinct clustering patterns that effectively separated the experimental groups (Figures 3, 4). In liver tissue, the pronounced separation of the high-dose processed iron powder group (TH) from the control group (CK) indicates substantial metabolic perturbations, primarily affecting energy metabolism, amino acid homeostasis, and redox balance. In contrast, both low- and high-dose QWTX groups exhibited metabolic profiles closely resembling those of controls, suggesting minimal hepatotoxic impact and supporting the hepatic safety of QWTX. This observation was further validated by LC-MS data, in which the high-dose QWTX group (FH) showed clear separation along the first principal component (PC1), indicative of a distinct pharmacological signature, while exhibiting only minor deviations along PC2—unlike the acetaminophen (APAP) group, which displayed significant displacement along PC2, a pattern typically associated with xenobiotic-induced stress and toxicity.

In renal tissue, QWTX induced dose-dependent metabolic modulation. The low-dose group (FL) showed notable metabolic divergence from controls, potentially reflecting early therapeutic engagement or adaptive physiological responses. While both high-dose QWTX (FH) and high-dose processed iron (TH) groups exhibited modest metabolic changes, these were considerably less pronounced than the extensive disruptions caused by APAP. Importantly, the contrasting trajectories of FH and APAP along PC2 in cationic mode suggest fundamentally different mechanisms of action: APAP-induced nephrotoxicity is likely mediated through oxidative stress, mitochondrial dysfunction, and glutathione depletion following metabolic activation, whereas QWTX appears to modulate renal metabolism through alternative, less toxic pathways.

By integrating untargeted metabolomics with biochemical and oxidative stress markers, this study provides a systems-level understanding of the metabolic networks influenced by QWTX, processed iron powder, and APAP in rat liver and kidney tissues. The findings demonstrate that these interventions do not elicit isolated metabolic changes but instead induce interconnected perturbations centered on impaired energy metabolism, increased oxidative stress, dysregulated nitrogen metabolism, and disturbances in neuroendocrine signaling. These integrated responses highlight the complexity of drug-induced metabolic effects and underscore the relative safety and distinct biological impact of QWTX compared to both iron exposure and a known hepatorenal toxin.

### 4.3 Hepatorenal metabolic adaptation to energy stress induced by QWTX and processed iron powder treatment

Metabolic reprogramming—particularly of energy metabolism—emerges as a central mechanism underlying the pathophysiological responses to the tested interventions. In the liver, both high-dose QWTX (FH) and high-dose processed iron powder (TH) led to the accumulation of citrate and isocitrate, accompanied by elevated fumarate and reduced malate levels. These metabolic shifts collectively suggest disruptions in the tricarboxylic acid (TCA) cycle, specifically at the level of isocitrate dehydrogenase (IDH) and fumarase (Gafson et al., 2025; Srere, 1974), indicating impaired flux through key steps of mitochondrial respiration. This blockade in the TCA cycle compromises ATP production and triggers cellular energy stress.

This energy deficit is further substantiated at the transcriptional level by qPCR analysis. In the TH group, significant downregulation of ATP5A1—a critical subunit of mitochondrial ATP synthase—and SDHA, a core enzyme of complex II in the electron transport chain—confirms impaired oxidative phosphorylation (Xu and Li, 2016), consistent with the observed TCA cycle dysregulation. Additionally, key components of the mTOR signaling pathway, MTOR and RPTOR, were markedly downregulated. This suppression aligns with activation of the “AMPK/SIRT1–TSC1–mTOR” regulatory axis, a well-conserved signaling cascade that governs cellular energy homeostasis. Under energy-deprived conditions—characterized by low ATP and a high AMP/ATP ratio—PRKAA1 (encoding the catalytic subunit of AMPK) and SIRT1 are upregulated. These two energy sensors act synergistically to activate the TSC1/TSC2 complex (Towler and Hardie, 2007), which inhibits RHEB-mediated activation of mTORC1. Consequently, mTORC1-driven anabolic processes such as protein synthesis are suppressed, while catabolic pathways like autophagy are enhanced to restore energy balance (Cohen et al., 2004). The significant upregulation of PRKAA1 and SIRT1 in the liver confirms the activation of this adaptive stress response pathway in response to metabolic challenge.

In parallel, the marked reduction in circulating and hepatic branched-chain amino acids (BCAAs)—leucine, isoleucine, and valine—along with increased levels of pyridoxal phosphate (PLP), the active form of vitamin B6, points to enhanced BCAA catabolism (Hutson, 2001). BCAA degradation is initiated by branched-chain amino acid transaminase (BCAT), which transfers the amino group to α-ketoglutarate to form branched-chain α-keto acids (BCKAs), a reaction dependent on PLP as a cofactor. Subsequently, BCKAs undergo irreversible oxidative decarboxylation by the branched-chain α-keto acid dehydrogenase complex (BCKDH), a rate-limiting step that channels carbon skeletons into the TCA cycle as acetyl-CoA or succinyl-CoA for ATP generation (Dimou et al., 2022). The E1α subunit of BCKDH, encoded by BCKDHA, is a key determinant of flux through this pathway (White et al., 2018).

qPCR analysis revealed significant upregulation of BCAT2 and BCKDHA in kidney tissue, particularly in the TH group, indicating that the kidney not only expresses the complete BCAA catabolic machinery but actively upregulates it under metabolic stress. This finding provides a mechanistic explanation for the observed systemic depletion of BCAAs and reveals a coordinated hepatorenal metabolic adaptation to energy deficiency. Under such conditions—similar to those induced by nutrient deprivation or cold stress—the liver initiates BCAA transamination, while the kidney acts as a “metabolic partner,” amplifying the oxidative degradation of BCKAs to generate energy substrates. This inter-organ collaboration reflects a strategic reallocation of nitrogen-rich amino acids from anabolic (protein synthesis) to catabolic (energy production) utilization, thereby compensating for impaired mitochondrial ATP synthesis.

Together, these findings illustrate a highly plastic, system-wide metabolic reprogramming response to energy stress. The liver and kidney engage in a division of metabolic labor, jointly enhancing BCAA catabolism to sustain energy homeostasis. This adaptive synergy underscores the body’s capacity for integrated metabolic regulation across organs in response to pharmacological or physiological challenges.

### 4.4 Dynamic imbalance between oxidative stress and antioxidant defense

Oxidative stress and the failure of antioxidant defense systems represent a central mechanism underlying the metabolic dysfunction observed in both liver and kidney tissues. Under physiological conditions, redox homeostasis is maintained through robust antioxidant systems, primarily the glutathione (GSH)-dependent pathway. However, mitochondrial impairment and metabolic disruption induced by high-dose treatments—particularly high-dose QWTX (FH) and processed iron powder (TH)—trigger excessive reactive oxygen species (ROS) production, overwhelming cellular defenses and initiating a self-amplifying cycle of oxidative damage (Tse et al., 2016).

In the liver, this imbalance is evidenced by elevated oxidized glutathione (GSSG) and a significantly increased GSSG/GSH ratio—classical markers of oxidative stress—alongside depletion of lipoic acid, a potent endogenous antioxidant (Joo and Sadikot, 2012; Sentellas et al., 2014). These findings indicate a shift toward a pro-oxidant intracellular environment. Although the suppression of pro-inflammatory prostaglandins (e.g., PGE_2_ and PGD_2_) via arachidonic acid metabolism suggests partial anti-inflammatory effects, this does not offset the prevailing oxidative burden.

The body mounts several compensatory responses to counteract ROS accumulation. Notably, γ-glutamylcysteine—an intermediate in *de novo* GSH synthesis—accumulates, reflecting upregulated glutamate–cysteine ligase (GCL) activity and an attempt to boost GSH production (Ferguson and Bridge, 2016). Concurrently, the GABA shunt is activated, converting glutamate to succinate via GABA, thereby bypassing NADH-generating steps in the TCA cycle and potentially supplying carbon skeletons for GSH synthesis (Ansari et al., 2021). However, this adaptive response is critically limited by cysteine deficiency, particularly in the FH and APAP groups. As the rate-limiting substrate for GSH synthesis, cysteine scarcity directly constrains the regeneration of functional GSH, undermining the entire antioxidant system (Yin et al., 2016).

Even more consequential is the inhibition of the pentose phosphate pathway (PPP), as indicated by the accumulation of glucose-6-phosphate and reduced levels of 6-phosphogluconate (Fuentes-Lemus et al., 2023). This suggests suppressed activity of glucose-6-phosphate dehydrogenase (G6PD) and 6-phosphogluconate dehydrogenase (6PGD)—the two key NADPH-producing enzymes. Since NADPH is essential for glutathione reductase (GR) to recycle GSSG back to GSH, PPP inhibition severs the redox regeneration cycle. This creates a cascade collapse in which cysteine deficiency restricts the *de novo* synthesis of glutathione (GSH), while concurrent suppression of the pentose phosphate pathway limits the availability of NADPH—the essential reducing equivalent required for glutathione reductase to regenerate GSH from its oxidized form (GSSG) (Njälsson and Norgren, 2005). As a result, the cellular capacity to maintain reduced GSH pools is severely compromised, impairing the antioxidant system’s ability to neutralize reactive oxygen species. This failure culminates in uncontrolled ROS accumulation, driving widespread oxidative damage, including lipid peroxidation, protein oxidation, and disruption of cellular membrane integrity.

This progression is confirmed by significantly elevated malondialdehyde (MDA) levels in the FH group—direct evidence of severe lipid peroxidation (Pirinccioglu et al., 2010). MDA elevation, combined with lipoic acid depletion and impaired GSH dynamics, positions the high-dose QWTX group in a state of fulminant oxidative stress, where endogenous defenses are overwhelmed.

In contrast, lower-intensity challenges—such as low-dose QWTX (FL) and both doses of processed iron powder (TL, TH)—elicit more effective antioxidant responses. GSH levels increase, and superoxide dismutase (SOD) activity is enhanced across iron-treated groups. SOD catalyzes the dismutation of superoxide (O^2-^) into H_2_O_2_, which is then detoxified by catalase or GPx, forming the first line of ROS defense (Younus, 2018). This coordinated upregulation suggests a successful adaptive response under moderate stress.

However, under high-dose QWTX, this compensation fails. A key contributor to the ROS surge is the upregulation of xanthine dehydrogenase (XDH) in the liver. During energy crisis, ATP degradation leads to the accumulation of hypoxanthine and xanthine—substrates for XDH (Wang et al., 2019; Zhao et al., 2024). XDH catalyzes their oxidation to uric acid, but in doing so generates substantial amounts of O^2-^ and H_2_O_2_ (Nishino et al., 2008). Thus, XDH induction not only reflects accelerated purine catabolism and worsening energy deficit but also acts as a “bypass” source of ROS, further fueling oxidative damage.

In the kidney, elevated flavin adenine dinucleotide (FAD) levels suggest compensatory activation of mitochondrial β-oxidation and the TCA cycle, likely an attempt to sustain ATP production via enhanced electron transport chain (ETC) flux (Olsen et al., 2016). However, this effort occurs against a backdrop of systemic energy deficiency and fails to prevent redox imbalance, especially when antioxidant regeneration is already compromised.

Collectively, these findings reveal a hierarchical failure of redox homeostasis: from initial mitochondrial ROS overproduction, through impaired antioxidant synthesis and regeneration, to irreversible oxidative damage. The transition from adaptive compensation to systemic collapse is determined by treatment intensity: low-dose stimuli activate protective pathways, while high-dose QWTX exceeds the system’s compensatory capacity, triggering a vicious cycle of energy depletion, purine degradation, XDH activation, ROS burst, and antioxidant failure.

### 4.5 Global disruption of nitrogen metabolism reflects systemic hepato-renal axis dysfunction

Disruption of nitrogen metabolism represents a central pathological mechanism in hepato-renal axis dysfunction, characterized by a critical imbalance in urea cycle regulation and amino acid homeostasis. This study reveals a profound “synthesis-excretion” disconnect under drug intervention, wherein enhanced hepatic urea production is uncoupled from impaired renal urea elimination—resulting in systemic nitrogen retention despite active detoxification efforts.

In the high-dose QWTX (FH) group, elevated hepatic citrulline and urea levels indicate upregulated urea cycle activity. Citrulline accumulation reflects increased metabolic flux through the cycle, suggesting a compensatory hepatic response to ammonia overload (Husson et al., 2003). This adaptive mechanism aims to maintain nitrogen homeostasis through enhanced ammonia detoxification in the liver—the primary site of urea synthesis.

However, this hepatic compensation is effectively nullified by concurrent renal dysfunction. In kidney tissue, urea levels were significantly reduced, while argininosuccinate—a key intermediate produced by argininosuccinate synthase (ASS1) and cleaved by argininosuccinate lyase (ASL)—accumulated markedly. This pattern suggests impaired ASL activity or a downstream block in substrate processing (Nagamani et al., 2012), disrupting local arginine regeneration and nitrogen handling. Although the kidney does not perform the complete urea cycle, it expresses ASS1 and ASL to support intrarenal arginine synthesis and nitrogen balance, underscoring its metabolic role beyond filtration.

This enzymatic dysregulation coincides with structural and functional proximal tubular injury, as evidenced by synchronized elevations in NGAL and Cystatin C—established biomarkers of tubular damage (Gharishvandi et al., 2015). The proximal tubules are responsible for reabsorbing 40%–50% of filtered urea to preserve medullary osmolarity. Tubular injury compromises this reabsorptive capacity, paradoxically reducing urea excretion efficiency despite increased delivery. Consequently, urinary nitrogen elimination is impaired, leading to systemic retention of blood urea nitrogen (BUN) across all treatment groups.

Critically, this nitrogen retention arises not from excessive protein catabolism or severe glomerular filtration rate (GFR) decline, but from excretory dysfunction driven by tubular impairment—a phenomenon we define as functional nitrogen retention. This highlights a systemic failure of the hepato-renal axis, where liver and kidney fail to coordinate in nitrogen waste management, despite intact or even enhanced synthetic capacity in the liver.

Beyond urea cycle disruption, widespread disturbances in amino acid metabolism further destabilize systemic homeostasis. Notably, activation of the methionine cycle is indicated by elevated 2-oxobutyrate and spermidine levels. 2-oxobutyrate is a byproduct of cysteine synthesis via the transsulfuration pathway, implicating increased methionine flux; spermidine elevation reflects heightened polyamine synthesis, which consumes S-adenosylmethionine (SAM)—the primary methyl donor for epigenetic, protein, and lipid modifications (Albers, 2009). These changes suggest a cellular attempt to sustain proliferation and stress adaptation under metabolic pressure.

Yet this adaptive activation comes at a steep cost: severe depletion of L-methionine and key exogenous methyl donors—choline and betaine—in the kidney. These compounds are essential for remethylating homocysteine to regenerate methionine via the BHMT pathway, thereby maintaining the methyl pool (Ueland, 2011). Their depletion indicates exhaustion of methyl reserves, threatening the integrity of methylation reactions critical for gene regulation, neurotransmission, and membrane integrity—foreshadowing a collapse in methylation homeostasis.

Concurrently, tryptophan metabolism is profoundly suppressed in the kidney, evidenced by reduced levels of downstream metabolites such as indole-3-acetic acid (IAA) and 5-methoxyindoleacetic acid (5-MIAA). Tryptophan serves as a precursor for serotonin, melatonin, and microbiota-derived indoles, many of which undergo hepatic conjugation before renal excretion (Xue et al., 2023). These metabolites are not only potential uremic toxins but also signaling molecules in the gut-kidney axis, modulating inflammation and oxidative stress. Their decline points to diminished activity of renal metabolic enzymes—including monoamine oxidases and UDP-glucuronosyltransferases (UGTs)—and may contribute to deficits in neurotransmitter precursors, potentially affecting both central and peripheral nervous system function (Boer et al., 2016).

In sum, drug-induced hepato-renal toxicity is not merely a consequence of isolated organ damage but reflects a systemic collapse of interorgan metabolic coordination. The core pathology involves a triad of disruptions: (1) a hepato-renal disconnect in urea handling leading to functional nitrogen retention; (2) methyl pool exhaustion due to imbalanced methionine metabolism; and (3) neurotransmitter precursor deficiency arising from suppressed tryptophan catabolism. Together, these interconnected perturbations demonstrate the fragility of cross-organ metabolic networks and underscore the importance of the “organ axis” framework in elucidating complex toxicological mechanisms.

### 4.6 Extensive neuroendocrine dysregulation reflects systemic collapse of regulatory networks

The neuroendocrine system serves as both a sensor and integrator of metabolic stress, and its widespread dysregulation represents not only a consequence of systemic metabolic collapse but also a critical amplifier of multi-organ injury. This study reveals a profound, multi-layered disruption across central and peripheral neuroendocrine pathways—spanning neurotransmitter synthesis, circadian regulation, stress response, and hormonal homeostasis—culminating in a self-sustaining cycle of physiological deterioration.

At the core of this dysregulation is a critical deficit in catecholamine precursor availability, evidenced by the consistent reduction of L-DOPA in kidney tissue across all treatment groups. L-DOPA, synthesized by tyrosine hydroxylase (TH), is the essential precursor for dopamine, norepinephrine, and epinephrine (Nagatsu and Sawada, 2009). While primarily associated with the central nervous system, renal proximal tubular epithelial cells also express the full catecholamine synthetic machinery and locally produce dopamine to regulate renal hemodynamics, sodium excretion, and inflammation (Bianchi et al., 2003). The depletion of L-DOPA thus signifies a bottleneck in both systemic sympathetic tone and intrarenal dopaminergic signaling, potentially impairing vascular resistance, cardiac output, and fluid-electrolyte balance—key determinants of organ perfusion and metabolic stability.

This dopaminergic deficit is compounded by parallel suppression of the tryptophan-serotonin-melatonin axis. Tryptophan is the precursor for serotonin (5-HT) and melatonin, two pivotal regulators of neural, metabolic, and immune function. The observed decline in downstream metabolites indicates impaired conversion, likely due to reduced enzymatic activity (e.g., tryptophan hydroxylase, AANAT/ASMT) in the context of renal metabolic dysfunction. Serotonin deficiency may disrupt central mood regulation, gastrointestinal motility, and platelet function, while also impairing liver regeneration (Mammadova-Bach et al., 2018). More critically, melatonin levels were significantly reduced, undermining its essential roles beyond circadian control: as a potent antioxidant, anti-inflammatory agent, and mitochondrial protector (Ahmad et al., 2023). The loss of melatonin not only desynchronizes metabolic rhythms—such as insulin sensitivity and lipid oxidation—but also weakens cellular defense against oxidative stress, accelerating tissue injury and impairing recovery.

These neurotransmitter and neuromodulator deficits converge on the hypothalamic-pituitary-adrenal (HPA) axis, the central coordinator of the body’s stress response. In the liver, elevated corticosterone and cortisone levels indicate systemic HPA axis activation, reflecting a compensatory effort to mobilize energy via gluconeogenesis, lipolysis, and immunosuppression (Karaca et al., 2021). However, this adaptive response becomes maladaptive in the high-dose QWTX (FH) group, where cortisol synthesis is impaired despite upstream stimulation—evidenced by accumulation of steroid precursors and reduced terminal products. This “upstream activation, downstream blockade” points to dysfunction in key steroidogenic enzymes, such as 11β-hydroxylase, potentially due to drug-induced adrenal or peripheral tissue toxicity (Janssen, 2022). The resulting glucocorticoid insufficiency creates a dangerous mismatch: high stress demand meets inadequate hormonal supply, undermining metabolic adaptation and immune regulation.

Renal steroid metabolism reveals further complexity. While most groups exhibited reduced cortisol and aldosterone—suggesting adrenocortical suppression or altered intrarenal hormone handling—the FH group paradoxically showed elevated renal cortisol. This apparent contradiction likely reflects not compensation but tissue-specific dysregulation, where cortisol accumulation promotes insulin resistance, sodium retention, and pro-fibrotic signaling in already injured tubules, thereby exacerbating renal dysfunction.

Even more concerning is the broad suppression of sex hormone pathways. Marked reductions in estrone (E1) and dihydrotestosterone (DHT) analogs indicate disrupted androgen and estrogen metabolism (Mozar et al., 2022). Beyond reproductive roles, sex hormones are vital for tissue integrity: testosterone supports protein anabolism, muscle maintenance, and tubular cell proliferation; estrogens exert anti-inflammatory, antioxidant, and vasoprotective effects that promote repair and mitigate fibrosis (Sciarra et al., 2023). Their deficiency compromises regenerative capacity, delays recovery from injury, and destabilizes electrolyte and vascular homeostasis—further weakening the resilience of multiple organ systems.

In sum, the observed neuroendocrine disruption forms a self-reinforcing network of failure: impaired neurotransmitter synthesis reduces autonomic and circadian control; HPA axis dysfunction creates energetic and immune imbalance; and sex hormone deficiency undermines tissue repair. These axes do not act in isolation but interact synergistically—neural signals modulate endocrine output, hormones regulate metabolic enzymes, and metabolic stress feeds back onto neural circuits. The result is a transition from localized metabolic adaptation to global physiological collapse.

This integrated dysregulation underscores the necessity of evaluating neuroendocrine endpoints in toxicological studies. Far from being secondary effects, these changes represent central drivers of systemic toxicity, highlighting the value of a holistic, axis-based framework in mechanistic risk assessment.

### 4.7 Iron chelation and herbal synergy in the traditional formula QWTX

A major safety concern associated with metallic ingredients—such as processed iron powder in traditional formulations—is their potential to induce ferroptosis via iron overload. This regulated form of cell death is driven by intracellular labile iron, particularly Fe^2+^, which catalyzes the Fenton reaction, generating excessive reactive oxygen species (ROS) that initiate lipid peroxidation and membrane damage (Li et al., 2020). In this context, the polyphenolic composition of Terminalia chebula, a key component in the processing of iron within QWTX, emerges as a critical protective agent. Our LC-MS analysis identified gallic acid, ellagic acid, and galloyl tannins—compounds well-documented for their strong iron-chelating properties. By forming stable complexes with free iron, these polyphenols likely reduce the pool of redox-active Fe^2+^, thereby suppressing Fenton-driven ROS production and mitigating oxidative injury (Wang C. et al., 2024).

This iron-sequestering mechanism is further supported by the observed enhancement of endogenous antioxidant defenses in the low-dose groups: elevated SOD activity and increased GSH levels indicate an adaptive upregulation of cellular redox buffering capacity in response to mild oxidative challenge. Concurrently, these polyphenols exhibit potent anti-inflammatory activity, evidenced by significant reductions in pro-inflammatory prostaglandins—including PGE_2_ and PGD_2_. The dual action of iron chelation and inflammation suppression illustrates a key principle of traditional processing: the transformation of potentially toxic components into therapeutically beneficial forms through synergistic herbal interactions.

Importantly, the dose-dependent effects observed in this study highlight the delicate balance between efficacy and toxicity. While high-dose treatments (TH and FH) triggered oxidative stress and metabolic disruption—likely due to pharmacological overload that overwhelms protective mechanisms—the low-dose complete formulation (FL) demonstrated superior therapeutic outcomes. Specifically, the FL group exhibited more pronounced and statistically robust suppression of inflammatory mediators (PGE_2_, PGJ_2_, PGD_2_, and 11-epi-PGF_2_α) compared to the TL group (low-dose processed iron without full herbal matrix), underscoring the advantage of the full QWTX composition.

This superiority reflects herbal synergy: the integrated pharmacological actions of multiple ingredients that collectively enhance detoxification, metabolic regulation, and tissue protection. For instance, the significantly reduced bile acid levels in both liver and kidney tissues of the FL group suggest improved hepatobiliary excretion and metabolic homeostasis. This effect is likely mediated by the combined contributions of multiple herbal components in QWTX: *Trogopterus Dung*, traditionally used to promote blood circulation and alleviate stasis; *Flos Carthami*, which has documented hepatoprotective properties; *Herba Dracocephali Tangutici*, long applied in the treatment of hepatitis and gastritis; *Costustoot*, known for its regulatory effects on digestion; and *Zong Bu Ju Hua*, recognized for its choleretic and anti-inflammatory actions in hepatic and gastric disorders (Wang S. et al., 2024). Together, these ingredients may synergistically enhance bile acid homeostasis and support coordinated regulation of liver and gastrointestinal function.

Together, these components orchestrate a multi-target, systems-level modulation of digestive function, inflammatory signaling, and redox balance—going beyond additive effects to achieve coordinated physiological regulation.

Critically, *Gypsum Rubrum* (processed gypsum) may further contribute by resolving stasis and supporting detoxification in gastrointestinal contexts, reinforcing the formula’s holistic action. The integration of these diverse yet complementary agents exemplifies the core principles of Tibetan medicine: “toxicity reduction through processing” and “therapeutic synergy through formulation.”

Our findings thus provide a modern mechanistic framework for these ancient practices. The processing of iron with *Terminalia chebula* transforms a potentially harmful metal into a biologically regulated component, while the full QWTX formula achieves superior efficacy through network pharmacology—modulating oxidative stress, inflammation, and energy metabolism in a coordinated, organ-system-wide manner. This study underscores that the therapeutic power of traditional polypharmacy lies not in single ingredients, but in their intelligent combination—a principle increasingly validated by metabolomics, redox biology, and systems medicine.

### 4.8 Study limitations

While this study provides valuable insights into the acute toxicological profile of processed iron powder and the traditional formula QWTX, several critical limitations must be acknowledged, highlighting the need for further investigation to fully assess its safety and therapeutic potential.

First, the 7-day exposure period—although sufficient to capture acute responses such as renal hemorrhage, oxidative stress, and metabolic disruption—does not reflect the long-term safety implications of mineral-based formulations. Traditional medicines containing metallic components carry inherent risks of cumulative tissue deposition and progressive organ damage with prolonged use. Therefore, extended toxicological evaluations, including sub-chronic (90-day) and chronic (6- to 12-month) studies at clinically relevant doses, are essential to determine the long-term consequences of repeated administration. Such studies would enable assessment of time-dependent accumulation of iron and other minerals in target organs, the evolution of metabolic disturbances into structural pathology, and the sustainability of hepatic and renal compensatory mechanisms.

Furthermore, the use of supratherapeutic doses (10× and 40× clinical equivalents), while strategically justified for amplifying toxicological signals in preclinical screening, limits direct translational applicability. These high doses may induce effects not representative of clinical exposure, particularly given interspecies differences in drug metabolism, distribution, and elimination. Moreover, the short duration of the study precludes the detection of delayed or cumulative toxicities that may only emerge after prolonged or repeated dosing—such as fibrosis, endocrine disruption, or functional decline due to chronic oxidative stress.

Although untargeted metabolomic profiling revealed significant perturbations in key pathways—including nitrogen metabolism, redox balance, and neuroendocrine signaling—the underlying molecular mechanisms and regulatory networks remain incompletely characterized. The observed metabolic shifts likely arise from complex interactions between drug components and cellular signaling cascades, but without deeper mechanistic exploration, causality cannot be established. Future studies integrating multi-omics approaches—particularly transcriptomics and proteomics—are necessary to map the upstream regulators (e.g., Nrf2, NF-κB, and PPARs) and downstream effectors driving these changes, thereby constructing a systems-level understanding of QWTX-induced biological responses.

Another important consideration is the complexity of QWTX’s mineral composition. While processed iron powder is the primary metallic component, the inclusion of Gypsum Rubrum—composed mainly of CaCO_3_ and CaSO_4_·2H_2_O—introduces potential calcium-related safety concerns. Although calcium is not classified as a heavy metal, excessive intake can lead to hypercalcemia, nephrocalcinosis, and vascular calcification, particularly in individuals with impaired excretory function. Thus, the contribution of calcium to the overall mineral burden and its potential synergistic or additive effects with iron warrant dedicated investigation, especially in the context of long-term use.

Despite these safety considerations, QWTX demonstrated promising therapeutic potential, particularly in the context of liver disease. Notably, even though its direct antioxidant capacity was relatively lower than that of processed iron powder alone, QWTX exhibited superior anti-inflammatory activity, likely mediated by a constellation of bioactive compounds such as ellagic acid, gallic acid, gentiopicrin, gallotannin, and apocynin (Chao et al., 2010; Ghosh et al., 2012; Xiao et al., 2013; Bai et al., 2021; Zhang et al., 2023). These constituents are known to modulate inflammatory signaling pathways—including NF-κB and MAPK—and may also influence gut-liver axis communication and immune cell activation. This multi-target, synergistic action underscores the formula’s ability to simultaneously regulate oxidative stress, inflammation, and metabolic homeostasis, reflecting the core principle of herbal polypharmacy.

In conclusion, this study establishes a foundational understanding of the acute toxicological mechanisms associated with processed iron and QWTX, revealing both risks and therapeutic benefits. However, it also emphasizes the necessity of comprehensive long-term safety assessments that account for cumulative exposure, mineral interactions, and delayed effects. Future research should combine extended exposure models with mechanistic multi-omics analyses and formulation-specific risk evaluation to ensure the safe and rational clinical application of QWTX. Only through such integrated approaches can the full spectrum of benefits and risks of complex mineral-herbal formulations be accurately defined.

## 5 Conclusion

This comprehensive study elucidates the complex and interconnected mechanisms underlying the toxicological profiles of Qiwei Tiexie Pill (QWTX) and its processed iron powder component through an integrated approach combining histopathological, biochemical, and multi-platform metabolomic analyses. Acute exposure to these agents induces significant metabolic perturbations that converge on four core physiological domains: energy metabolism, redox homeostasis, nitrogen handling, and neuroendocrine regulation. These systems do not operate in isolation; rather, their dysregulation forms a tightly interwoven network of metabolic stress that underlies organ dysfunction.

While both processed iron powder and QWTX induce dose-dependent metabolic changes, QWTX demonstrates a markedly improved safety profile compared to processed iron powder alone. Notably, high-dose processed iron powder causes moderate renal histopathological alterations—characterized by vascular changes and hemorrhage—and induces profound metabolic disturbances in both liver and kidney, including TCA cycle disruption, oxidative stress, and purine metabolism dysregulation. In contrast, QWTX, even at high doses, preserves renal architecture and mitigates iron-associated nephrotoxicity, likely due to the protective effects of its herbal constituents. This suggests that the full formulation achieves not only therapeutic synergy but also toxicity attenuation through herb-metal interactions.

The pathophysiological mechanisms revealed in this study extend beyond isolated organ damage to systemic inter-organ dysregulation. A key finding is the “hepato-renal disconnect” in nitrogen metabolism: while the liver upregulates urea cycle activity in response to metabolic stress, concurrent renal tubular injury impairs urea reabsorption and excretion, leading to functional nitrogen retention. This uncoupling highlights a failure in cross-organ coordination within the hepatorenal axis. Furthermore, a cascade failure in antioxidant defense is observed under high-dose QWTX, driven by cysteine deficiency and suppression of the pentose phosphate pathway, which collectively impair glutathione regeneration and culminate in lipid peroxidation. Concomitant neuroendocrine dysregulation—including depletion of catecholamine and serotonin precursors, HPA axis imbalance, and suppression of sex hormone pathways—indicates a systemic collapse of regulatory networks that amplifies multi-organ injury.

These findings carry significant clinical implications. The dose-dependent nature of metabolic and histopathological effects underscores the importance of strict adherence to recommended dosing regimens in clinical practice. The extensive metabolic reprogramming observed, particularly in individuals with pre-existing liver or kidney conditions, warrants comprehensive monitoring of hepatic, renal, and redox parameters during treatment. The superior safety and enhanced anti-inflammatory efficacy of the complete QWTX formulation, compared to processed iron powder alone, validate the traditional principles of mineral processing and herbal synergy in Tibetan medicine.

Nonetheless, several limitations must be acknowledged. The 7-day exposure period captures acute responses but does not reflect the risks of long-term use, such as cumulative mineral deposition or progressive organ damage. The supratherapeutic doses used, while appropriate for toxicological screening, may not fully represent clinical exposure scenarios. Moreover, the contribution of non-iron mineral components—particularly calcium from *Gypsum Rubrum*—to overall mineral burden and potential nephrocalcinosis or vascular calcification risks remains uncharacterized and requires dedicated investigation.

Future studies should prioritize long-term sub-chronic and chronic toxicity assessments at clinically relevant doses, complemented by multi-omics integration (transcriptomics, proteomics, and metabolomics) to map upstream regulatory pathways and causal mechanisms. Such research will be essential to fully define the safety window of QWTX and to enable its rational, evidence-based clinical application.

In summary, this work provides a systems-level understanding of the acute toxicological mechanisms of QWTX and processed iron powder, revealing both therapeutic promise and potential risks. It establishes a critical foundation for future investigations aimed at ensuring the safe and effective use of this traditional Tibetan formulation in modern clinical settings.

## Data Availability

The original contributions presented in the study are included in the article/Supplementary Material, further inquiries can be directed to the corresponding authors.
