# Supplementary material for: Comprehensive safety assessment of Qiwei Tiexie Pill: integrating histopathological, biochemical, and metabolomic analyses in a rat model
Source: Front Pharmacol. 2025 Sep 3;16:1567316. doi: 10.3389/fphar.2025.1567316 (PMC12441281; doi:10.3389/fphar.2025.1567316)
Supplement: Supplementary file 1 [file Table1.docx]

Supplementary Material

# Supplementary Table

## Supplementary Table 1 The primer sequences are as follows.

| **Gene** | **Forward (5′-3′)** | **Reverse (5′-3′)** |
| --- | --- | --- |
| β-actin | GGACCTGACAGACTACCTCA | GTTGCCAATAGTGATGACCT |
| Sdha | AGACGTTTGACAGGGGAATG | TCATCAATCCGCACCTTGTA |
| PRKAA1 | AAAGTGAAGGTGGGCAAGCAC | ATTTTCCCGACCACGTCCAG |
| Sirt1 | CTGTTTCCTGTGGGATACCTG | TCCACTGCACAGGCACATAC |
| Xdh | GCATCGCCAGCTCTCAGTAT | ACAAACTCACTGCGCTCGTA |
| Hprt1 | GTCCCAGCGTCGTGATTAGT | CTTGCCGCTGTCTTTTAGGC |
| Bckdha | CATCCCTGGATGACAAGCCC | CGGTACTGCTCAGAGGTTGG |
| Bcat2 | TGGCTGCAGCTATACTAGGAC | TGGAGTCTCCGGGGAAGTAG |
| Tsc1 | TAAACAGCTGAGGGGGAGGA | CGCACATGCTCCATCATTGG |
| Atp5a1 | TCCGAGAAGCTGCAAGGATG | TGGACCGCTTCTGACCAATC |
| Mtor | CTGCACTTGTTGTTGCCTCC | ATCTCCCTGGCTGCTCCTTA |
| Rptor | TACGACTGCTCCAATGCTGG | TGACCGTTGTCCTTCACGAG |

## Supplementary Table 1 Identification of chemical components in extracts from Qiwei Tiexi Pill

| **No.** | **Retention Time (min)** | **Experimental** | **Mass Error (ppm)** | **Adduct ions** | **Molecular formula** | **Proposed compound** | **MS/MS Fiagments** |
| --- | --- | --- | --- | --- | --- | --- | --- |
| 1 | 0.566 | 195.0547 | 18.6 | M+FA-H | C5H10O5 | aldehydo-D-ribose | 59.0165，75.0101，129.0244，159.0348，195.0511 |
| 2 | 0.612 | 481.0711 | 18.1 | M-H | C20H18O14 | 2,3-hexahydroxydiphenyl-D-glucose | 275.0252，301.0041，481.0696 |
| 3 | 0.67 | 337.0261 | 17.8 | M-H | C14H10O10 | ellagic acid | 119.0528，133.0693，163.0437，205.0550，249.0466， |
| 4 | 1.443 | 169.0173 | 17.9 | M-H | C7H6O5 | gallic acid | 79.0204，124.0186，125.0260，169.0171 |
| 5 | 2.823 | 669.1091 | 21.9 | M-H | C27H26O20 | Phyllanemblinin D | 249.0443，337.0255，669.1068 |
| 6 | 2.881 | 243.0569 | -38.6 | M-H | C14H12O4 | Oroselol | 125.0269，124.0186，169.0179，243.0558 |
| 7 | 3.042 | 483.0462 | 17.3 | M-H | C20H20O14 | Gallic acid-3-O-(6'-O-galloyl) glucoside | 125.0260，168.0096，169.0169，193.0181，211.0287 |
| 8 | 3.261 | 107.2002 | 17.3 | M+FA-H | C31H34O16 | crassirhizomoside A | 191.0603，707.1961 |
| 9 | 3.318 | 635.0982 | 14.5 | M-H | C27H24O18 | gallotannin | 169.0196，465.0788，635.1010 |
| 10 | 4.1 | 165.0596 | 25.6 | M-H | C9H10O3 | Apocynin | 72.9959，101.0416，119.0546，147.0489，165.0659 |
| 11 | 4.25 | 401.12135 | 30.9 | M+FA-H | C16H20O9 | Gentiopicrin | 96.9623，241.0082，401.1857，401.1204 |
| 12 | 4.25 | 623.174 | 19.6 | M+FA-H | C27H30O14 | Lespedin | 284.0386，299.0619，623.1772 |
| 13 | 4.48 | 345.1631 | 22.1 | M+FA-H | C15H24O6 | Patrinoside-aglycone | 75.0106，113.0270，345.1623 |
| 14 | 4.905 | 421.1235 | -27.7 | M+FA-H | C16H24O10 | 6-O-methylcatalpol | 59.0148，137.0269，145.0320，163.0428，215.0598 |
| 15 | 5.114 | 582.272 | 19 | M-H | C34H37N3O6 | MEGxp0_000365 | 5.115，5.192，5.271 |
| 16 | 6.023 | 309.2154 | 26.9 | M+FA-H | C17H28O2 | cedryl acetate | 71.0520，109.0683，157.0894，171.1074，183.1087 |
| 17 | 6.588 | 305.1822 | 20.7 | M+FA-H | C17H24O2 | Falcarindiol | 79.0593，125.1010，135.0849，205.1635，249.1563 |
| 18 | 7.239 | 391.2947 | 23..8 | M-H, M+FA-H | C24H40O4 | Deoxycholic Acid | 345.2898，391.2945 |
| 19 | 7.764 | 467.3283 | 24.8 | M-H | C30H44O4 | glabrolide | 239.2044，421.3192，467.3265 |
| 20 | 7.914 | 295.2352 | 24.8 | M-H | C18H32O3 | Vernolic acid | 171.1065，195.1435，277.2235，295.2342 |
| 21 | 8.052 | 515.3426 | 9.3 | M+FA-H | C30H46O5 | Chinova acid | 191.0645，253.0989，265.1540，279.2391，311.1819 |
| 22 | 8.259 | 297.2507 | 24.2 | M-H | C18H34O3 | Nouracid CS 80 | 297.2525，297.1594 |
| 23 | 8.259 | 235.1738 | 14.5 | M-H | C15H24O2 | Cyperolone | 134.8997，205.1715，233.1630，235.1790 |
| 25 | 8.316 | 559.3748 | 19.3 | M+FA-H | C32H50O5 | Alisol B monoacetate | 44.9994，247.0351，265.1506，513.3160，531.3794 |
| 26 | 9.135 | 464.2998 | -4.3 | M-H | C26H43NO6 | GCH | 231.1460，232.1491，464.3031 |
| 27 | 10.179 | 281.2556 | 24.8 | M-H | C18H34O2 | 2-Octadecenoic acid | 281.2546 |
| 28 | 10.766 | 383.1999 | 35.3 | M-H | C23H28O5 | 2,6-dihydroxy-4-[(e)-7-hydroxy-3,7-dimethyl-octa-2-enyloxy] benzophenone | 100.9371，116.9321，339.2028，365.1887，383.2004 |
| 29 | 4.459 | 251.1643 | 0.4 | M+H | C15H22O3 | Viscic acid | 67.0543，91.0541，105.0685，131.0836，173.1320 |
| 30 | 5.824 | 235.1686 | -0.7 | M+H | C15H22O2 | ISOPETASOL | 81.0694，93.0694，105.0688，131.0644，161.0953 |
| 31 | 4.297 | 253.179 | -3.3 | M+H | C15H24O3 | ilicic acid | 79.0540,81.0684,105.0687,147.1147,175.1461 |
| 32 | 5.882 | 235.1691 | -0.9 | M+H | C15H22O2 | Curcumenol | 67.0545,93.0703,105.0697,131.0850,161.0969 |
| 33 | 7.218 | 233.1531 | -2.2 | M+H | C15H20O2 | Costunolide | 55.0547,93.0701,105.0691,145.1006,173.0961 |
| 34 | 7.253 | 231.137 | -4.3 | M+H-H2O | C15H20O3 | Carabron | 77.0374，95.0851，128.0616，143.0831，183.1315 |
| 35 | 8.071 | 471.3452 | -3.6 | M+H-H2O | C15H22O3 | Hydroxyvalerenic Acid | 189.1625,235.1677,425.3383,471.3417 |
| 36 | 8.071 | 471.3452 | -3.6 | M+H | C30H46O4 | enoxolone | 189.1625，235.1677，425.3383，471.3417 |
